# Genome-wide analyses identify 30 loci associated with obsessive–compulsive disorder

**DOI:** 10.1038/s41588-025-02189-z

**Published:** 2025-05-13

**Authors:** Nora I. Strom, Zachary F. Gerring, Marco Galimberti, Dongmei Yu, Matthew W. Halvorsen, Abdel Abdellaoui, Cristina Rodriguez-Fontenla, Julia M. Sealock, Tim Bigdeli, Jonathan R. Coleman, Behrang Mahjani, Jackson G. Thorp, Katharina Bey, Christie L. Burton, Jurjen J. Luykx, Gwyneth Zai, Silvia Alemany, Christine Andre, Kathleen D. Askland, Julia Bäckman, Nerisa Banaj, Cristina Barlassina, Judith Becker Nissen, O. Joseph Bienvenu, Donald Black, Michael H. Bloch, Sigrid Børte, Rosa Bosch, Michael Breen, Brian P. Brennan, Helena Brentani, Joseph D. Buxbaum, Jonas Bybjerg-Grauholm, Enda M. Byrne, Judit Cabana-Dominguez, Beatriz Camarena, Adrian Camarena, Carolina Cappi, Angel Carracedo, Miguel Casas, Maria Cristina Cavallini, Valentina Ciullo, Edwin H. Cook, Jesse Crosby, Bernadette A. Cullen, Elles J. De Schipper, Richard Delorme, Srdjan Djurovic, Jason A. Elias, Xavier Estivill, Martha J. Falkenstein, Bengt T. Fundin, Lauryn Garner, Christina Gironda, Fernando S. Goes, Marco A. Grados, Jakob Grove, Wei Guo, Jan Haavik, Kristen Hagen, Kelly Harrington, Alexandra Havdahl, Kira D. Höffler, Ana G. Hounie, Donald Hucks, Christina Hultman, Magdalena Janecka, Eric Jenike, Elinor K. Karlsson, Kara Kelley, Julia Klawohn, Janice E. Krasnow, Kristi Krebs, Christoph Lange, Nuria Lanzagorta, Daniel Levey, Kerstin Lindblad-Toh, Fabio Macciardi, Brion Maher, Brittany Mathes, Evonne McArthur, Nathaniel McGregor, Nicole C. McLaughlin, Sandra Meier, Euripedes C. Miguel, Maureen Mulhern, Paul S. Nestadt, Erika L. Nurmi, Kevin S. O’Connell, Lisa Osiecki, Olga Therese Ousdal, Teemu Palviainen, Nancy L. Pedersen, Fabrizio Piras, Federica Piras, Sriramya Potluri, Raquel Rabionet, Alfredo Ramirez, Scott Rauch, Abraham Reichenberg, Mark A. Riddle, Stephan Ripke, Maria C. Rosário, Aline S. Sampaio, Miriam A. Schiele, Anne Heidi Skogholt, Laura G. Sloofman, Jan Smit, María Soler Artigas, Laurent F. Thomas, Eric Tifft, Homero Vallada, Nathanial van Kirk, Jeremy Veenstra-VanderWeele, Nienke N. Vulink, Christopher P. Walker, Ying Wang, Jens R. Wendland, Bendik S. Winsvold, Yin Yao, Hang Zhou, Estonian Biobank, Andres Metspalu, Tõnu Esko, Reedik Mägi, Mari Nelis, Georgi Hudjashov, 23andMe Inc., Chris German, Arpana Agrawal, Pino Alonso, Götz Berberich, Kathleen K. Bucholz, Cynthia M. Bulik, Danielle Cath, Damiaan Denys, Valsamma Eapen, Howard Edenberg, Peter Falkai, Thomas V. Fernandez, Abby J. Fyer, J. M. Gaziano, Dan A. Geller, Hans J. Grabe, Benjamin D. Greenberg, Gregory L. Hanna, Ian B. Hickie, David M. Hougaard, Norbert Kathmann, James Kennedy, Dongbing Lai, Mikael Landén, Stéphanie Le Hellard, Marion Leboyer, Christine Lochner, James T. McCracken, Sarah E. Medland, Preben B. Mortensen, Benjamin M. Neale, Humberto Nicolini, Merete Nordentoft, Michele Pato, Carlos Pato, David L. Pauls, John Piacentini, Christopher Pittenger, Danielle Posthuma, Josep Antoni Ramos-Quiroga, Steven A. Rasmussen, Margaret A. Richter, David R. Rosenberg, Stephan Ruhrmann, Jack F. Samuels, Sven Sandin, Paul Sandor, Gianfranco Spalletta, Dan J. Stein, S. Evelyn Stewart, Eric A. Storch, Barbara E. Stranger, Maurizio Turiel, Thomas Werge, Ole A. Andreassen, Anders D. Børglum, Susanne Walitza, Kristian Hveem, Bjarne K. Hansen, Christian Rück, Nicholas G. Martin, Lili Milani, Ole Mors, Ted Reichborn-Kjennerud, Marta Ribasés, Gerd Kvale, David Mataix-Cols, Katharina Domschke, Edna Grünblatt, Michael Wagner, John-Anker Zwart, Gerome Breen, Gerald Nestadt, Jaakko Kaprio, Paul D. Arnold, Dorothy E. Grice, James A. Knowles, Helga Ask, Karin J. Verweij, Lea K. Davis, Dirk J. Smit, James J. Crowley, Jeremiah M. Scharf, Murray B. Stein, Joel Gelernter, Carol A. Mathews, Eske M. Derks, Manuel Mattheisen

**Affiliations:** 1 Department of Psychology, Humboldt-Universität zu Berlin, Berlin, Germany; 2 Department of Psychiatric Phenomics and Genomics (IPPG), Ludwig-Maximilians University Munich, Munich, Germany; 3 Centre for Psychiatry Research, Department of Clinical Neuroscience, Karolinska Institutet and Stockholm Health Services, Region Stockholm, Stockholm, Sweden; 4 Department of Biomedicine, Aarhus University, Aarhus, Denmark; 5 University Hospital of Psychiatry and Psychotherapy, University of Bern, Bern, Switzerland; 6 Department of Mental Health and Neuroscience, Translational Neurogenomics, QIMR Berghofer Medical Research Institute, Brisbane, Queensland Australia; 7 Department of Population Health and Immunity, Healthy Development and Ageing, Walter and Eliza Hall Institute of Medical Research, Melbourne, Victoria Australia; 8 Department of Psychiatry, Human Genetics, Yale University, New Haven, CT USA; 9 VA Connecticut Healthcare System, West Haven, CT USA; 10 Department of Center for Genomic Medicine, Psychiatric and Neurodevelopmental Genetics Unit, Massachusetts General Hospital, Boston, MA USA; 11 Stanley Center for Psychiatric Research, Broad Institute of MIT and Harvard, Cambridge, MA USA; 12 Department of Genetics, University of North Carolina at Chapel Hill, Chapel Hill, NC USA; 13 Department of Psychiatry, Amsterdam University Medical Centers, University of Amsterdam, Amsterdam, the Netherlands; 14 Center for Research in Molecular Medicine and Chronic Diseases (CiMUS), Genomics and Bioinformatics, University of Santiago de Compostela, Santiago de Compostela, Spain; 15 Grupo de Medicina Xenómica, Genetics, Instituto de Investigación Sanitaria de Santiago de Compostela (FIDIS), Santiago de Compostela, Spain; 16 Vanderbilt Genetics Institute, Vanderbilt University, Nashville, TN USA; 17 Department of Psychiatry and Behavioral Sciences, SUNY Downstate Health Sciences University, Brooklyn, NY USA; 18 VA NY Harbor Healthcare System, Brooklyn, NY USA; 19 Social, Genetic and Developmental Psychiatry Centre, Institute of Psychiatry, Psychology and Neuroscience, King’s College London, London, UK; 20 National Institute for Health and Care Research Maudsley Biomedical Research Centre, South London and Maudsley NHS Trust, London, UK; 21 Department of Psychiatry, Icahn School of Medicine at Mount Sinai, New York, NY USA; 22 Department of Medical Epidemiology and Biostatistics, Karolinska Institutet, Stockholm, Sweden; 23 Mental Health and Neuroscience Program, QIMR Berghofer Medical Research Institute, Brisbane, Queensland Australia; 24 Faculty of Medicine, School of Biomedical Sciences, University of Queensland, Brisbane, Queensland Australia; 25 Department of Psychiatry and Psychotherapy, University Hospital Bonn, Bonn, Germany; 26 Department of Neurosciences and Mental Health, Hospital for Sick Children, Toronto, Ontario Canada; 27 Department of Psychiatry, Brain University Medical Center Utrecht, Utrecht, the Netherlands; 28 Second Opinion Outpatient Clinic, GGNet, Warnsveld, the Netherlands; 29 Molecular Brain Science Department, Campbell Family Mental Health Research Institute, Centre for Addiction and Mental Health, Toronto, Ontario Canada; 30 Department of Psychiatry, University of Toronto, Toronto, Ontario Canada; 31 Psychiatric Genetics Unit, Group of Psychiatry, Mental Health and Addiction, Vall d’Hebron Research Institute, Universitat Autònoma de Barcelona, Barcelona, Spain; 32 Department of Mental Health, Hospital Universitari Vall d’Hebron, Barcelona, Spain; 33 Biomedical Network Research Centre on Mental Health (CIBERSAM), Madrid, Spain; 34 Obsessive–Compulsive Disorder Institute, McLean Hospital, Belmont, MA USA; 35 Department of Psychiatry and Behavioural Neurosciences, McMaster University, Hamilton, Hamilton, Ontario Canada; 36 Laboratory of Neuropsychiatry, IRCCS Santa Lucia Foundation, Rome, Italy; 37 Department of Health Sciences, University of Milano, Milano, Italy; 38 Department of Child and Adolescent Psychiatry, Aarhus University Hospital, Psychiatry, Aarhus, Denmark; 39 Institute of Clinical Medicine, Health, Aarhus University, Aarhus, Denmark; 40 Department of Psychiatry and Behavioral Sciences, General Hospital Psychiatry, Johns Hopkins University School of Medicine, Baltimore, MD USA; 41 Departments of Roy J. and Lucille A. Carver College of Medicine, University of Iowa, Iowa City, IA USA; 42 Department of Child Study Center and Psychiatry, Yale University, New Haven, CT USA; 43 Department of Research and Innovation, Division of Clinical Neuroscience, Oslo University Hospital, Oslo, Norway; 44 Department of Public Health and Nursing, Faculty of Medicine and Health Sciences, HUNT Center for Molecular and Clinical Epidemiology, Norwegian University of Science and Technology (NTNU), Trondheim, Norway; 45 Department of Medical Genetics, Oslo University Hospital, Oslo, Norway; 46 Department of Child and Adolescent Mental Health, Hospital Sant Joan de Déu, Esplugues de Llobregat, Spain; 47 Instituto de Salut Carlos III, Centro de Investigación Biomédica en Red de Salut Mental (CIBERSAM), Madrid, Spain; 48 Seaver Autism Center for Research and Treatment, Icahn School of Medicine at Mount Sinai, New York, NY USA; 49 The Mindich Child Health and Development Institute, Icahn School of Medicine at Mount Sinai, New York, NY USA; 50 Department of Psychiatry, Harvard Medical School, Boston, MA USA; 51 Department of Psychiatry, Universidade de São Paulo, São Paulo, Brazil; 52 Department for Congenital Disorders, Statens Serum Institut, Copenhagen, Denmark; 53 Child Health Research Centre, University of Queensland, Brisbane, Queensland Australia; 54 Pharmacogenetics Department, Investigaciones Clínicas, Instituto Nacional de Psiquiatría Ramon de la Fuente Muñiz, Mexico City, México; 55 Department of Surgery, Duke University, Durham, NC USA; 56 Department of Psychiatry, Icahn School of Medicine at Mount Sinai Hospital, New York, NY USA; 57 Department of Psychiatry, University of São Paulo, São Paulo, Brazil; 58 CiMUS, Genomics and Bioinformatics Group, University of Santiago de Compostela, Santiago de Compostela, Spain; 59 Galician Foundation of Genomic Medicine, Grupo de Medicina Xenómica, Instituto de Investigación Sanitaria de Santiago (IDIS), Santiago de Compostela, Spain; 60 Medicina Genómica, Centro de Investigación Biomédica en Red, Enfermedades Raras (CIBERER), Santiago de Compostela, Spain; 61 Programa MIND Escoles, Hospital Sant Joan de Déu, Esplugues de Llobregat, Spain; 62 Departamento de Psiquiatría y Medicina Legal, Universitat Autònoma de Barcelona, Bellaterra, Spain; 63 Department of Psychiatry, Ospedale San Raffaele, Milano, Italy; 64 Department of Psychiatry, University of Illinois Chicago, Chicago, IL USA; 65 Department of Psychiatry and Behavioral Sciences, Johns Hopkins Medical Institutions, Baltimore, MD USA; 66 Department of Mental Health, Bloomberg School of Public Health, Baltimore, MD USA; 67 Child and Adolesccent Psychiatry Department, APHP, Paris, France; 68 Department of Clinical Science, University of Bergen, Bergen, Norway; 69 Psychiatry, McLean Hospital OCDI, Harvard Medical School, Belmont, MA USA; 70 Adult Psychological Services, CBTeam LLC, Lexington, MA USA; 71 qGenomics (Quantitative Genomics Laboratories), Esplugues de Llobregat, Spain; 72 Department of Medical Epidemiology and Biostatistics, Center for Eating Disorders Innovation, Karolinska Institutet, Stockholm, Sweden; 73 Department of Psychiatry, Johns Hopkins University, Baltimore, MD USA; 74 Department of Psychiatry and Behavioral Sciences, Child and Adolescent Psychiatry, Johns Hopkins University, Baltimore, MD USA; 75 The Lundbeck Foundation Initiative for Integrative Psychiatric Research, iPSYCH, Aarhus, Denmark; 76 Center for Genomics and Personalized Medicine, Aarhus, Denmark; 77 Bioinformatics Research Centre, Aarhus, Denmark; 78 Genetic Epidemiology Research Branch, National Institute of Mental Health, Bethesda, MD USA; 79 Department of Biomedicine, University of Bergen, Bergen, Norway; 80 Bergen Center for Brain Plasticity, Division of Psychiatry, Haukeland University Hospital, Bergen, Norway; 81 Department of Psychiatry, Møre og Romsdal Hospital Trust, Molde, Norway; 82 Bergen Center for Brain Plasticity, Psychiatry, Haukeland University Hospital, Bergen, Norway; 83 Department of Mental Health, Norwegian University for Science and Technology, Trondheim, Norway; 84 Million Veteran Program (MVP) Coordinating Center, VA Boston Healthcare System, Boston, MA USA; 85 Department of Psychiatry, Boston University Chobanian and Avedisian School of Medicine, Boston, MA USA; 86 PsychGen Centre for Genetic Epidemiology and Mental Health, Norwegian Institute of Public Health, Oslo, Norway; 87 Nic Waals Institute, Lovisenberg Diaconal Hospital, Oslo, Norway; 88 Bergen Center for Brain Plasticity, Haukeland University Hospital, Bergen, Norway; 89 Department of Medical Genetics, Dr. Einar Martens Research Group for Biological Psychiatry, Haukeland University Hospital, Bergen, Norway; 90 Department of Medicine, Genetic Medicine, Vanderbilt University Medical Center, Nashville, TN USA; 91 Department of Child and Adolescent Psychiatry, NYU Grossman School of Medicine, New York, NY USA; 92 Department of Bioinformatics and Integrative Biology, University of Massachusetts Medical School, Worcester, MA USA; 93 Department of Vertebrate Genomics, Broad Institute of MIT and Harvard, Cambridge, MA USA; 94 Department of Medicine, MSB Medical School Berlin, Berlin, Germany; 95 Department of Psychiatry and Behavioral Sciences, Johns Hopkins University, Baltimore, MD USA; 96 Estonian Genome Centre, Institute of Genomics, University of Tartu, Tartu, Estonia; 97 Department of Biostatistics, T.H. Chan School of Public Health, Boston, MA USA; 98 Department of Medicine, Channing Division of Network Medicine, Brigham and Women’s Hospital, Boston, MA USA; 99 Research Department, Grupo Medico Carracci, Mexico City, Mexico; 100 Department of Psychiatry, Yale University, West Haven, CT USA; 101 Office of Research and Development, United States Department of Veterans Affairs, West Haven, CT USA; 102 Department of Medical Biochemistry and Microbiology, Uppsala University, Uppsala, Sweden; 103 SciLifeLab, Uppsala University, Uppsala, Sweden; 104 Department of Psychiatry, University of California, Irvine, Irvine, CA USA; 105 Department of Mental Health, Johns Hopkins Bloomberg School of Public Health, Baltimore, MD USA; 106 Vanderbilt Genetics Institute, Vanderbilt University Medical Center, Nashville, TN USA; 107 COBRE Center for Neuromodulation, Butler Hospital, Providence, RI USA; 108 Department of Psychiatry and Human Behavior, Alpert Medical School, Brown University, Providence, RI USA; 109 Butler Hospital, Providence, RI USA; 110 Department of Psychiatry, Dalhousie University, Halifax, Nova Scotia Canada; 111 Department of Psychiatry, Faculdade de Medicina, Universidade de São Paulo, São Paulo, Brazil; 112 Department of Psychiatry and Behavioral Science, Johns Hopkins University, Baltimore, MD USA; 113 Department of Psychiatry and Biobehavioral Sciences, Division of Child and Adolescent Psychiatry, University of California, Los Angeles, Los Angeles, CA USA; 114 Department of Clinical Medicine, Division of Mental Health and Addiction, Oslo University Hospital, Oslo, Norway; 115 NORMENT, University of Oslo, Oslo, Norway; 116 Department of Psychiatry, Massachusetts General Hospital, Boston, MA USA; 117 Psychiatric and Neurodevelopmental Genetics Unit, Harvard Medical School, Boston, MA USA; 118 Department of Biomedicine, Haukeland University Hospital, Bergen, Norway; 119 Institute for Molecular Medicine Finland (FIMM), University of Helsinki, Helsinki, Finland; 120 Department of Clinical Neuroscience and Neurorehabilitation, Neuropsychiatry Laboratory, IRCCS Santa Lucia Foundation, Rome, Italy; 121 Department of Genetics, Microbiology and Statistics, IBUB, Universitat de Barcelona, Barcelona, Spain; 122 CIBERER, Centro de Investigación Biomédica en Red, Madrid, Spain; 123 Department of Human Molecular Genetics, Institut de Recerca Sant Joan de Déu, Barcelona, Spain; 124 Department of Psychiatry and Psychotherapy, Division of Neurogenetics and Molecular Psychiatry, Faculty of Medicine and University Hospital Cologne, University of Cologne, Cologne, Germany; 125 Department of Neurodegenerative Diseases and Geriatric Psychiatry, Medical Faculty, University Hospital Bonn, Bonn, Germany; 126 DZNE Bonn, German Center for Neurodegenerative Diseases (DZNE), Bonn, Germany; 127 Department of Psychiatry and Glenn Biggs Institute for Alzheimer’s and Neurodegenerative Diseases, UT Health San Antonio, San Antonio, TX USA; 128 Cologne Excellence Cluster for Stress Responses in Ageing-associated Diseases (CECAD), University of Cologne, Cologne, Germany; 129 Department of Psychiatry, McLean Hospital, Harvard Medical School, Belmont, MA USA; 130 Department of Mental Disorders, Norwegian Institute of Public Health, New York, NY USA; 131 Department of Psychiatry and Behavioral Sciences, Child and Adolescent, Johns Hopkins University School of Medicine, Baltimore, MD USA; 132 Department of Psychiatry and Psychotherapy, Charité—Universitätsmedizin, Berlin, Germany; 133 Stanley Center for Psychiatric Research, Broad Institute of Harvard and MIT, Cambridge, MA USA; 134 Site Berlin–Potsdam, German Center for Mental Health (DZPG), Berlin, Germany; 135 Department of Psychiatry, Child and Adolescent Psychiatry Unit (UPIA), Federal University of São Paulo (UNIFESP), São Paulo, Brazil; 136 Department of Neurosciences and Mental Health, Medical School, Federal University of Bahia, Salvador, Brazil; 137 Department of Psychiatry and Psychotherapy, Faculty of Medicine, University of Freiburg, Medical Center—University of Freiburg, Freiburg, Germany; 138 Department of Public Health and Nursing, HUNT Center for Molecular and Clinical Epidemiology, Trondheim, Norway; 139 Department of Psychiatry, Faculty of Medicine, Locaion VUmc, Amsterdam UMC, University of Amsterdam, Amsterdam, the Netherlands; 140 Department of Genetics, Microbiology, and Statistics, Faculty of Biology, Universitat de Barcelona, Barcelona, Spain; 141 Department of Clinical and Molecular Medicine, NTNU, Trondheim, Norway; 142 Department of Public Health and Nursing, K.G. Jebsen Center for Genetic Epidemiology, NTNU, Trondheim, Norway; 143 BioCore, Bioinformatics Core Facility, NTNU, Trondheim, Norway; 144 Clinic of Laboratory Medicine, St. Olavs Hospital, Trondheim University Hospital, Trondheim, Norway; 145 Department of Molecular Medicine and Surgery, CMM, Karolinska Institutet, Stockholm, Sweden; 146 OCD Institute, Division of Depression and Anxiety, McLean Hospital, Belmont, MA USA; 147 Department of Psychiatry, Harvard Medical School, Belmont, MA USA; 148 Department of Psychiatry, Division of Child and Adolescent Psychiatry, Columbia University, New York, NY USA; 149 Department of Child and Adolescent Psychiatry, New York State Psychiatric Institute, New York, NY USA; 150 Department of Psychiatry, Amsterdam UMC, University of Amsterdam, Amsterdam, the Netherlands; 151 Human Genetics Institute of New Jersey, Rutgers University, Piscataway, NJ USA; 152 Department of Neurology, the Johns Hopkins University School of Medicine, Baltimore, MD USA; 153 Laboratory of Clinical Science, NIMH Intramural Research Program, Bethesda, MD USA; 154 Department of Neurology, Oslo University Hospital, Oslo, Norway; 155 HUNT Center for Molecular and Clinical Epidemiology, Department of Public Health and Nursing, Faculty of Medicine and Health Sciences, NTNU, Trondheim, Norway; 156 Department of Computional Biology, Institute of Life Science, Fudan University, Fudan, China; 157 Department of Psychiatry, Yale School of Medicine, New Haven, CT USA; 158 Department of Psychiatry, Veterans Affairs Connecticut Healthcare System, West Haven, CT USA; 159 Section of Biomedical Informatics and Data Science, Yale School of Medicine, New Haven, CT USA; 160 Department of Psychiatry, Washington University School of Medicine, Saint Louis, MO USA; 161 Department of Psychiatry, OCD Clinical and Research Unit, Bellvitge Hospital, Barcelona, Spain; 162 Department of Clinical Sciences, University of Barcelona, Barcelona, Spain; 163 Department of Psychiatry and Mental Health, Bellvitge Biomedical Research Institute IDIBELLL, Barcelona, Spain; 164 CIBERSAM, Mental Health Network Biomedical Research Center, Madrid, Spain; 165 Psychosomatic Department, Windach Hospital of Neurobehavioural Research and Therapy, Windach, Germany; 166 Department of Psychiatry, Washington University School of Medicine, St Louis, MO USA; 167 Department of Psychiatry, University of North Carolina at Chapel Hill, Chapel Hill, NC USA; 168 Department of Nutrition, University of North Carolina at Chapel Hill, Chapel Hill, NC USA; 169 Departments of Rijksuniversiteit Groningen and Psychiatry, University Medical Center Groningen, Groningen, the Netherlands; 170 Department of Specialized Training, Drenthe Mental Health Care Institute, Groningen, the Netherlands; 171 Department of Psychiatry, Institute of the Royal Netherlands Academy of Arts and Sciences (NIN-KNAW), Amsterdam, the Netherlands; 172 Discipline of Psychiatry and Mental Health, School of Clinical Medicine, UNSW, Sydney, New South Wales Australia; 173 Academic Unit of Child Psychiatry South-West Sydney, South-West Sydney Clinical School, SWSLHD and Ingham Institute, Sydney, New South Wales Australia; 174 Department of Biochemistry and Molecular Biology, Indiana University School of Medicine, Indianapolis, IN USA; 175 Department of Psychiatry and Psychotherapy, University Hospital LMU, Munich, Germany; 176 Department of Psychiatry, Max Planck Institute, Munich, Germany; 177 Child Study Center and Psychiatry, Yale School of Medicine, New Haven, CT USA; 178 Department of Psychiatry, New York State Psychiatric Institute, New York, NY USA; 179 Department of Psychiatry, Columbia University Medical Center, New York, NY USA; 180 Department of Medicine, VA Boston Healthcare System, Boston, MA USA; 181 Department of Medicine, Mass General Brigham, Boston, MA USA; 182 Department of Psychiatry, Child Psychiatry, Massachusetts General Hospital, Boston, MA USA; 183 Department of Psychiatry and Psychotherapy, University Medicine Greifswald, Greifswald, Germany; 184 COBRE Center on Neuromodulation, Butler Hospital, Providence, RI USA; 185 Center for Neurorestoration and Neurotechnology, VA Providence Healthcare System, Providence, RI USA; 186 Department of Psychiatry and Human Behavior, Alpert Medical School, Brown University, Providence, RI USA; 187 Department of Psychiatry, Child and Adolescent Psychiatry, University of Michigan, Ann Arbor, MI USA; 188 Brain and Mind Centre, the University of Sydney, Sydney, New South Wales Australia; 189 Department of Medical and Molecular Genetics, Indiana University School of Medicine, Indianapolis, IN USA; 190 Institute of Neuroscience and Physiology, Department of Psychiatry and Neurochemistry, University of Gothenburg, Gothenburg, Sweden; 191 Department of Addictology and Psychiatry, Université Paris-Est Créteil, AP-HP, Inserm, Paris, France; 192 Department of Psychiatry, SA MRC Unit on Risk and Resilience in Mental Disorders, Stellenbosch University, Stellenbosch, South Africa; 193 Department of Mental Health, Psychiatric Genetics, QIMR Berghofer Medical Research Institute, Brisbane, Queensland Australia; 194 National Centre for Register-based Research, Aarhus University, Aarhus, Denmark; 195 Centre for Integrated Register-based Research, Aarhus University, Aarhus, Denmark; 196 Analytic and Translational Genetics Unit, Massachusetts General Hospital, Boston, MA USA; 197 Department of Psychiatry, Psychiatry, Carracci Medical Group, Mexico City, México; 198 Psiquiatría, Instituto Nacional de Medicina Genómica, Mexico City, México; 199 Mental Health Center Copenhagen, Copenhagen Research Center for Mental Health, Mental Health Services in the Capital Region of Denmark, Copenhagen, Denmark; 200 Faculty of Health and Medical Sciences, Department of Clinical Medicine, University of Copenhagen, Copenhagen, Denmark; 201 Department of Psychiatry, Rutgers University, Piscataway, NJ USA; 202 Department of Psychiatry and Biobehavioral Sciences, Child and Adolescent Psychiatry, UCLA Semel Institute for Neuroscience and Human Behavior, Los Angeles, CA USA; 203 Department of Psychiatry, Yale University, New Haven, CT USA; 204 Department of Complex Trait Genetics, Vrije Universiteit Amsterdam, Center for Neurogenomics and Cognitive Research, Amsterdam Neuroscience, Amsterdam, the Netherlands; 205 Department of Child and Adolescent Psychiatric, Section Complex Trait Genetics, VU Medical Center Amsterdam, Amsterdam, the Netherlands; 206 Department of Psychiatry, Hospital Universitari Vall d’Hebron, Barcelona, Spain; 207 Group of Psychiatry, Mental Health and Addictions, Psychiatric Genetics Unit, Vall d’Hebron Research Institute, Barcelona, Spain; 208 CIBERSAM, Barcelona, Spain; 209 Department of Psychiatry and Forensic Medicine, Universitat Autònoma de Barcelona, Barcelona, Spain; 210 Department of Psychiatry, Sunnybrook Health Sciences Centre, Toronto, Ontario Canada; 211 Department of Psychiatry and Behavioral Neurosciences, Child and Adolescent Psychiatry, Wayne State University School of Medicine, Detroit, MI USA; 212 Department of Psychiatry and Psychotherapy, University of Cologne, Cologne, Germany; 213 Department of Psychiatry and Behavioral Sciences, the Johns Hopkins University School of Medicine, Baltimore, MD USA; 214 Department of Psychiatry and Behavioral Sciences, Division of Neuropsychiatry, Baylor College of Medicine, Houston, TX USA; 215 Department of Psychiatry and Neuroscience Institute, SAMRC Unit on Risk and Resilience in Mental Disorders, University of Cape Town, Cape Town, South Africa; 216 Department of Psychiatry, University of British Columbia, Vancouver, British Columbia Canada; 217 British Columbia Children’s Hospital Research Institute, Vancouver, British Columbia Canada; 218 British Columbia Mental Health and Substance Use Services Research Institute, Vancouver, British Columbia Canada; 219 Department of Psychiatry and Behavioral Sciences, Baylor College of Medicine, Houston, TX USA; 220 Department of Pharmacology, Northwestern University Feinberg School of Medicine, Chicago, IL USA; 221 Center for Genetic Medicine, Northwestern University Feinberg School of Medicine, Chicago, IL USA; 222 Department of Cardiology, University of Milan, Milan, Italy; 223 Institute of Biological Psychiatry, Mental Health Center Sct. Hans, Copenhagen University Hospital, Mental Health Services (RHP), Copenhagen, Denmark; 224 Institute of Clinical Medicine, University of Copenhagen, Copenhagen, Denmark; 225 Institute of Clinical Medicine, NORMENT Centre, University of Oslo, Oslo, Norway; 226 Division of Mental Health and Addiction, Center for Precision Psychiatry, Oslo University Hospital, Oslo, Norway; 227 The Lundbeck Foundation Initiative for Integrative Psychiatric Research, iPSYCH, Aarhus University, Aarhus, Denmark; 228 Center for Genomics and Personalized Medicine, Aarhus University, Aarhus, Denmark; 229 Department of Child and Adolescent Psychiatry and Psychotherapy, Psychiatric University Hospital Zurich (PUK), University of Zurich, Zürich, Switzerland; 230 Neuroscience Center Zurich, University of Zurich and the ETH Zurich, Zürich, Switzerland; 231 Zurich Center for Integrative Human Physiology, University of Zurich, Zürich, Switzerland; 232 HUNT Research Center, Department of Public Health and Nursing, Faculty of Medicine and Health Sciences, NTNU, Trondheim, Norway; 233 Department of Research, Innovation and Education, St. Olavs Hospital, Trondheim University Hospital, Trondheim, Norway; 234 Centre for Crisis Psychology, Psychology, University of Bergen, Bergen, Norway; 235 Department of Genetic Epidemiology, QIMR Berghofer Medical Research Institute, Brisbane, Queensland Australia; 236 Psychosis Research Unit, Psychiatry, Aarhus University Hospital, Aarhus, Denmark; 237 Department of Mental Disorders, Norwegian Institute of Public Health, Oslo, Norway; 238 Institute of Clinical Medicine, University of Oslo, Oslo, Norway; 239 Department of Clinical Psychology, Faculty of Psychology, University of Bergen, Bergen, Norway; 240 Partner Site Berlin, DZPG, Berlin, Germany; 241 Department of Neurodegenerative Diseases and Geriatric Psychiatry, University Hospital Bonn, Bonn, Germany; 242 DZNE, Bonn, Germany; 243 Department of Research and Innovation, Clinical Neuroscience, Oslo University Hospital and University of Oslo, Oslo, Norway; 244 Social, Genetic and Developmental Psychiatric Centre, Institute of Psychiatry, Psychology and Neuroscience, King’s College London, London, UK; 245 FIMM, University of Helsinki, Helsinki, Finland; 246 Department of Psychiatry, the Mathison Centre for Mental Health Research and Education, Cumming School of Medicine, University of Calgary, Calgary, Alberta Canada; 247 Program in Genetics and Genome Biology, Hospital for Sick Children, Toronto, Ontario Canada; 248 Department of Genetics, Human Genetics Institute of New Jersey, Rutgers University, Piscataway, NJ USA; 249 PsychGen Center for Genetic Epidemiology, Norwegian Institute of Public Health, Oslo, Norway; 250 PROMENTA Research Center, Department of Psychology, University of Oslo, Oslo, Norway; 251 Department of Medicine, Division of Genetic Medicine, Vanderbilt University Medical Center, Nashville, TN USA; 252 Department of Psychiatry, Amsterdam UMC location AMC, Amsterdam, the Netherlands; 253 Psychiatry Service, VA San Diego Healthcare System, San Diego, CA USA; 254 Department of Psychiatry and School of Public Health, University of California San Diego, La Jolla, CA USA; 255 Department of Psychiatry, Human Genetics (Psychiatry), Yale University School of Medicine, West Haven, CT USA; 256 Department of Psychiatry, Veterans Affairs Connecticut Healthcare Center, West Haven, CT USA; 257 Psychiatry and Genetics Institute, Evelyn F. and William L. Mc Knight Brain Institute, Center for OCD, Anxiety and Related Disorders, University of Florida, Gainesville, FL USA; 258 Department of Mental Health and Neuroscience, QIMR Berghofer, Brisbane, Queensland Australia; 259 Department of Community Health and Epidemiology and Faculty of Computer Science, Dalhousie University, Halifax, Nova Scotia Canada; 260 23andMe, Inc., Sunnyvale, CA USA

**Keywords:** Genome-wide association studies, Genetics research

## Abstract

Obsessive–compulsive disorder (OCD) affects ~1% of children and adults and is partly caused by genetic factors. We conducted a genome-wide association study (GWAS) meta-analysis combining 53,660 OCD cases and 2,044,417 controls and identified 30 independent genome-wide significant loci. Gene-based approaches identified 249 potential effector genes for OCD, with 25 of these classified as the most likely causal candidates, including *WDR6*, *DALRD3* and *CTNND1* and multiple genes in the major histocompatibility complex (MHC) region. We estimated that ~11,500 genetic variants explained 90% of OCD genetic heritability. OCD genetic risk was associated with excitatory neurons in the hippocampus and the cortex, along with D_1_ and D_2_ type dopamine receptor-containing medium spiny neurons. OCD genetic risk was shared with 65 of 112 additional phenotypes, including all the psychiatric disorders we examined. In particular, OCD shared genetic risk with anxiety, depression, anorexia nervosa and Tourette syndrome and was negatively associated with inflammatory bowel diseases, educational attainment and body mass index.

## Abstract (web summary)

Genome-wide analyses identify 30 independent loci associated with obsessive–compulsive disorder, highlighting genetic overlap with other psychiatric disorders and implicating putative effector genes and cell types contributing to its etiology.

## Main

OCD is a chronic psychiatric disorder that affects 1–3% of the population^1^ and is characterized by obsessions and compulsions that vary in type and severity and over time. OCD is responsible for profound personal and societal costs^2^, including increased risk of suicide^3^ and overall mortality^4^. OCD is moderately heritable; twin-based heritability estimates range between 27% and 47% in adults and between 45% and 65% in children^5–8^, with SNP-based heritability estimates between 28% and 37%^9–11^.

Two earlier OCD GWAS meta-analyses, both containing a subset of the data included in this analysis^12,13^, showed SNP-based heritabilities of 8.5% (assuming a 3% population prevalence) and 16% (assuming a 2% population prevalence). The first GWAS (*n*_cases_ = 14,140, *n*_controls_ = 562,117)^12^ found one genome-wide significant locus associated with OCD, while the second (*n*_cases_ = 37,015, *n*_controls_ = 948,616)^13^ identified 15 independent genome-wide significant loci. As with other complex traits, increased sample sizes are needed for a more comprehensive understanding of the underlying genetic etiology of OCD and its genetic relationships with related disorders.

The current study combines data from the two unpublished OCD GWASs described above and includes additional cohorts (~9,000 cases). This results in one of the largest and most well-powered GWAS of OCD so far, with a ~20-fold increase of OCD cases compared to the previously published OCD GWASs^10^. Based on the results from the meta-analysis, we conducted secondary analyses, including positional and functional fine-mapping of SNPs and genes, structural equation modeling to examine possible genetic differences in sample ascertainment across cohorts, protein and transcriptome-wide association analyses, single-cell enrichment and genetic correlations with other traits (Supplementary Fig. 1). Our results provide more detailed insight into the genetic underpinnings and biology of OCD.

## Results

### GWAS meta-analysis identifies 30 genome-wide significant loci

**Fig. 1:**
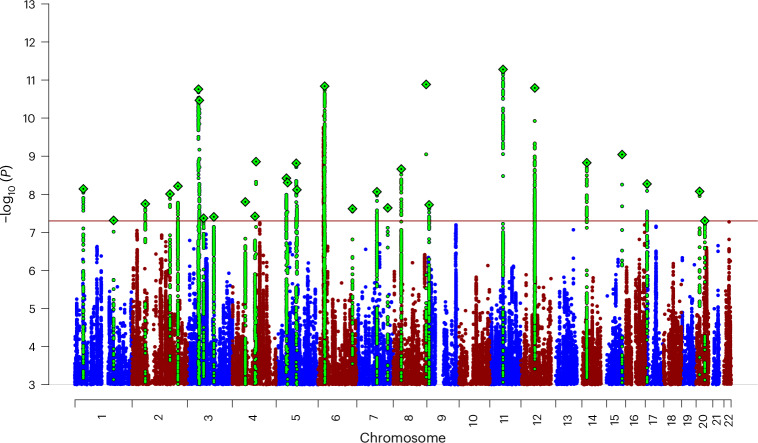
Manhattan plot of OCD GWAS meta-analysis. The *y* axis represents −log_10_ (*P* values) (two sided, not adjusted for multiple testing) for the association of variants with OCD using an inverse-variance-weighted fixed-effects model (*n*_cases_ = 53,660 and *n*_controls_ = 2,044,417). The *x* axis shows chromosomes 1–22. The horizontal red line represents the threshold for genome-wide significance ($P=5\times {10}^{-8})$. Index variants of genome-wide significant loci are highlighted as green diamonds.

We conducted a GWAS meta-analysis of 28 OCD case–control cohorts of European ancestry, comprising 53,660 cases and 2,044,417 controls (effective sample size, ~210,000 individuals). Ascertainment of cases varied across cohorts: OCD diagnosis was determined (1) by a healthcare professional in a clinical setting (18 cohorts, *n* = 9,089 cases), (2) from health records or biobanks (seven cohorts, *n* = 9,138 cases), (3) in a clinical setting or from health records with the additional characteristic that all OCD cases were primarily collected for another psychiatric disorder (three cohorts, *n* = 5,266 cases) or (4) by self-reported diagnosis in a consumer-based setting (23andMe, Inc., *n* = 30,167 cases). Cohort details, including phenotypic assessment, quality control and individual cohort GWAS analyses, are described in Supplementary Note 2 and Supplementary Table 1. We identified 30 independent (defined in Supplementary Note 3) loci among the 1,672 SNPs that exceeded the genome-wide threshold for significance ($P < 5{\times 10}^{-8}$; Manhattan plot in Fig. 1, regional association plots and forest plots in Supplementary Figs. 2–31 and a list of all independent genome-wide significant SNPs in Table 1 with additional details in Supplementary Tables 2 and 3). The independence of the 30 lead SNPs was subsequently validated using conditional and joint analysis (GCTA-COJO)^14^ (Supplementary Table 4). Analysis of the X chromosome, conducted in a subset of the data for which this information was available (23andMe), yielded no significant associations (Supplementary Note 4 and Supplementary Fig. 37e). Of the 15 genome-wide significant loci previously reported in preprints^12,13^, 13 were genome-wide significant in the current GWAS, with the remaining two showing suggestive significance ($P=5.23{\times 10}^{-8}\,{\rm{and}}\,{P}=2.2{\times 10}^{-7}$; Supplementary Table 5). Using MiXeR^15^, we estimated that approximately 11,500 (standard error of the effect estimate (s.e.) = 607) causal variants account for 90% of the OCD SNP-based heritability.

**Table 1:** Genome-wide-significant loci associated with OCD

SNP	Position	*P* value	OR	s.e.	A_1_/A_2_	FRQCA	FRQCO	INFO	*n* genes	Selection of other significant traits
rs78587207^a^	11q12.1	5.28 × 10^−12^	0.9522	0.0071	T/G	0.681	0.677	0.987	11	SCZ, well-being, neuroticism, educational attainment
rs13262595	8q24.3	1.31 × 10^−11^	0.9566	0.0066	G/A	0.552	0.561	0.989	2	SCZ, well-being, neuroticism, educational attainment
rs4990036^a^	6p21.33	1.45 × 10^−11^	0.9299	0.0108	T/C	0.119	0.123	0.985	118	SCZ, depression, blood cell count, lung function
rs10877425^a^	12q14.1	1.62 × 10^−11^	0.9526	0.0072	G/A	0.513	0.516	0.985	0	–
rs7626445^a^	3p21.31	1.74 × 10^−11^	0.9551	0.0068	T/C	0.647	0.654	0.994	32	Neuroticism, smoking, blood cell count, height
rs2564930^a^	3p21.1	3.41 × 10^−11^	0.9546	0.007	T/C	0.339	0.345	0.988	12	SCZ, neuroticism, blood cell count, BMI
rs4702^a^	15q26.1	9.07 × 10^−10^	1.0414	0.0066	G/A	0.455	0.449	0.984	5	SCZ, BP, MDD, risk-taking behavior
rs35518360^a^	4q24	1.39 × 10^−9^	1.0757	0.0121	T/A	0.0828	0.0756	0.947	1	SCZ, neuroticism, educational attainment, BMI
rs4904738	14q21.1	1.48 × 10^−9^	0.9605	0.0067	T/C	0.558	0.553	0.984	1	MDD
rs254779	5q14.3	1.53 × 10^−9^	0.9606	0.0067	T/C	0.419	0.421	0.988	4	Educational attainment, cognition, fat mass
rs2198140^a^	8p12	2.18 × 10^−9^	0.9590	0.007	T/C	0.496	0.513	0.979	1	Cognition
rs12516488^a^	5p12	3.79 × 10^−9^	1.0531	0.0088	G/A	0.825	0.826	0.994	1	Neuroticism, age at first birth, age at first sexual intercourse
rs3899258^a^	5q11.1	4.94 × 10^−9^	1.0509	0.0085	G/A	0.782	0.792	0.989	2	–
rs3027160	17p13.1	5.35 × 10^−9^	1.0497	0.0083	T/C	0.775	0.782	0.996	19	Sleep, height
rs203768	2q33.1	6.14 × 10^−9^	0.9513	0.0086	T/C	0.824	0.817	0.992	5	SCZ
rs11263940	1p34.3	7.23 × 10^−9^	0.9578	0.0074	T/C	0.689	0.69	0.991	0	Neuroticism, well-being
rs67839857^a^	5q14.3	7.63 × 10^−9^	1.0423	0.0072	G/A	0.692	0.691	0.994	0	–
rs1555466	20p11.23	8.42 × 10^−9^	1.0490	0.0083	T/C	0.218	0.224	0.996	0	Ease of skin tanning
rs9886111	7q21.13	8.59 × 10^−9^	0.9598	0.0071	G/C	0.701	0.711	0.992	2	–
rs9287859	2q24.3	9.83 × 10^−9^	0.9595	0.0072	G/A	0.39	0.39	0.994	1	–
rs2087319	4q12	1.59 × 10^−8^	0.9579	0.0076	C/A	0.74	0.744	0.968	6	Height, blood pressure
rs11125759^a^	2p16.1	1.79 × 10^−8^	0.9690	0.0071	G/A	0.569	0.556	0.991	1	BMI, sleep
rs6474628	9p23	1.89 × 10^−8^	1.0380	0.0066	T/G	0.579	0.585	0.999	0	–
rs11768238	7q33	2.28 × 10^−8^	0.9601	0.0073	G/A	0.661	0.661	0.998	2	Educational attainment, age at first sexual intercourse
rs9479138	6q25.1	2.41 × 10^−8^	1.0397	0.007	T/G	0.339	0.34	0.975	1	Educational attainment, age at first sexual intercourse, age at first birth, lung function
rs1567288	4q22.3	3.80 × 10^−8^	0.9643	0.0066	G/A	0.548	0.55	0.981	1	–
rs4831130	3q13.31	3.93 × 10^−8^	1.0427	0.0076	T/G	0.753	0.74	0.984	2	–
rs17718444	3p13	4.25 × 10^−8^	0.9622	0.007	T/C	0.323	0.32	0.984	2	Educational attainment, lung function, use of sun/UV protection
rs6660196^a^	1q24.1	4.86 × 10^−8^	1.0403	0.0072	T/G	0.638	0.642	0.989	1	Blood cell count
rs4931	20q13.12	5.00 × 10^−8^	0.9609	0.0073	C/A	0.278	0.279	0.993	5	Blood cell count, height, BMI

Shown are the lead SNP, the chromosome, base pair position on the genome, *P* value, effect estimate as an odds ratio (OR), s.e., effect allele and non-effect allele (A_1_ and A_2_), frequency of A_1_ in cases (FRQCA) and in controls (FRQCO), imputation quality score (INFO), number of genes in a region of 6.5 kb around the SNP (*n* genes) and a curated list of phenotypes that also showed a genome-wide significant association with this SNP (in one or more of the following four databases: CAUSALdb^90^, GenomeAtlas^52^, the NHGRI-EBI GWAS Catalog^91^, the IEU Open GWAS project^92^). If fewer than four traits are significant across all four databases, all four traits are shown. If more than five traits are significant across the databases, neuropsychiatric traits are prioritized (closely related traits are summarized into one trait category). For a full list of associations in the four databases, see Supplementary Table 18a–d. A more detailed list of the significant loci can be found in Supplementary Table 2. Abbreviations in the last column are SCZ, schizophrenia; BP, bipolar disorder; MDD, major depressive disorder; UV, ultraviolet.

^a^Previously identified GWAS hits for OCD (or SNPs in high linkage disequilibirum with a previously identified SNP).

No statistically significant heterogeneity was observed across individual cohorts for the 30 genome-wide significant loci, as assessed with Cochran’s *Q*-test (Supplementary Fig. 32), the *I*^2^ statistic and the genomic structural equation modeling (GenomicSEM) ${Q}_{{\rm{SNP}}}$ statistic^16^ (Supplementary Table 2). Genome-wide analyses of samples grouped by clinical, comorbid, biobank and 23andMe information (Supplementary Table 3 and Supplementary Figs. 33–37) showed evidence that sample ascertainment impacted results at a genome-wide scale, although not beyond what is observed with closely related psychiatric disorders^17,18^. We observed moderate to high genetic correlations across the subgroups (between 0.63, s.e. = 0.11 for biobanks and comorbid information and 0.92, s.e. = 0.07 for 23andMe and comorbid information; Supplementary Table 7) and a satisfactory fit for a one-factor GenomicSEM model (Supplementary Table 8 and Supplementary Fig. 39). A common factor GWAS based on the one-factor GenomicSEM model resulted in 20 significant loci, all of which were also significant in the primary GWAS (Supplementary Table 8 and Supplementary Fig. 40; analysis details in Supplementary Note 5). SNP heritability (assuming a 1% population prevalence) was 6.7% (s.e. = 0.3%), with slightly higher estimates for the clinical (${h}_{{\rm{SNP}}}^{2}$ = 16.4%, s.e. = 1.5%) and comorbid (${h}_{{\rm{SNP}}}^{2}$ = 13.3%, s.e. = 1.7%) subgroups (Supplementary Table 1).

### Gene-based findings

We prioritized putative risk genes for OCD using six positional and functional QTL gene-based mapping approaches. Positional mapping was performed with mBAT-combo^19^. Functional expression quantitative trait locus (eQTL) mapping was performed with transcriptome-wide association study (TWAS)^20^, using PsychENCODE gene expression weights^21^, and summary-based Mendelian randomization (SMR)^22^ using the whole-blood eQTLGen^23^ and MetaBrain^24^ datasets. Functional protein QTL mapping was done using a protein-wide association study (PWAS) of human brain protein expression panels^25^. Finally, we used the psychiatric omnilocus prioritization score (PsyOPS)^26^, which combines positional mapping with biological annotations, to further prioritize risk genes within genome-wide significant loci. We identified 207 significant genes (Bonferroni correction, *P* < 2.67 × 10^−^^6^) with mBAT-combo and 24 genes using TWAS (*P* < 4.76 × 10^−6^), 14 of which were conditionally independent. The SMR–eQTLGen analysis identified 39 significant risk genes (*P* < 4.28 × 10^−^^6^), and the SMR–MetaBrain analysis identified 14 risk genes (*P* < 9.23 × 10^−6^). The PWAS identified three significant genes (*P* < 3.39 × 10^−5^), while PsyOPS prioritized 29 genes. In total, 251 genes were significantly associated with OCD through at least one gene-based approach, and 48 were implicated by at least two methods (Methods, Supplementary Note 7 and Supplementary Tables 9–14).

**Fig. 2:**
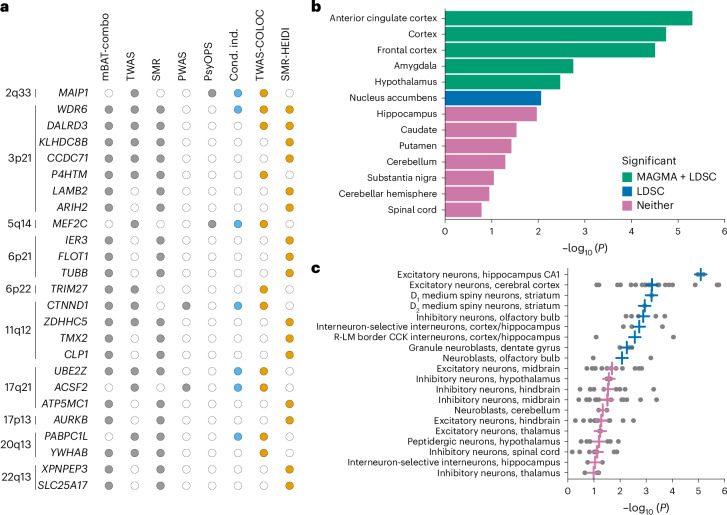
Gene-based, tissue and cell type enrichment analyses. **a**, List of 25 genes that were implicated in at least two of the five different gene-based tests (significance indicated by gray dots) and passed the TWAS colocalization and/or SMR-HEIDI filters (significance indicated by orange dots). Conditionally independent (cond. ind.) genes within each locus are indicated by blue dots. **b**, Enrichment of OCD GWAS signal in human brain-related tissues from GTEx (version 8). No significant enrichment was observed in the peripheral tissues (not included in the figure). The horizontal bar size represents the significance of the enrichment measured using the MAGMA gene set enrichment test or partitioned LDSC. **c**, Top 20 groups of brain cell types (*n* = 35 total tested) enriched with OCD GWAS signal using MAGMA. Dots represent −log_10_(*P* values) from MAGMA gene set enrichment tests of individual neuronal cell types from Zeisel et al.^30^. Vertical crosses represent the mean −log_10_(*P* value) observed for each brain cell type group. Blue crosses represent a significant enrichment of OCD GWAS signals (FDR across 35 groups, FDR < 0.05), while pink crosses indicate nonsignificant enrichment. Gray points represent the association (−log_10_(*P* value)) for each single cell cluster (‘level 5’ analysis defined by Zeisel et al.^30^) in a given cell type (for example, excitatory neurons, cerebral cortex). CCK, cholecystokinin-expressing; R-LM, stratum radiatum-stratum lacunosum-moleculare.

From the 48 genes implicated by at least two approaches, we prioritized likely causal genes for OCD using colocalization (TWAS-COLOC)^27,28^ and SMR–heterogeneity in dependent instruments (SMR-HEIDI)^22^ tests. Colocalization was used to identify significant TWAS associations for which the underlying GWAS and eQTL summary statistics are likely to share a single causal variant. Similarly, HEIDI was used to select SMR associations for which the same causal variant affects gene expression and trait variation. Of the 48 genes implicated by at least two gene-based tests, 25 were also significant in either the TWAS-COLOC or the SMR-HEIDI tests, suggesting causality (Fig. 2a). Only 2 of these 25 genes were prioritized by both TWAS-COLOC and SMR-HEIDI: *WDR6* (WD repeat domain 6) and *DALRD3* (DALR anticodon binding domain-containing 3). Another gene of interest, *CTNND1* (catenin δ1), was implicated by three of our five approaches (multivariate set-based association test (mBAT-combo), TWAS, PWAS) and showed evidence for colocalization. Only three genes were implicated in the PWAS; of these, *CTNND1* was the only gene also implicated in the TWAS. In the PWAS, downregulation of CTNND1 protein expression in the human dorsolateral prefrontal cortex (dlPFC) was significantly associated with OCD risk ($Z=-4.49,P=7.11\times {10}^{-6}$; Supplementary Table 13), consistent with the downregulation of *CTNND1* gene expression in the prefrontal cortex seen in the TWAS ($Z=-6.86,P=6.90\times {10}^{-12};$ Supplementary Table 10). For a discussion of the overlap between the gene findings with rare coding variants in OCD, see Supplementary Table 6 and Supplementary Note 7.

### Tissue and cell type enrichment analysis

After mapping significantly associated SNPs from the GWAS meta-analysis to likely causal genes, we explored which tissues or cell types showed enriched gene expression of OCD-associated genetic signals using a previously described approach^29^ on published human gene expression datasets from bulk tissue RNA-seq data from the Genotype–Tissue Expression (GTEx) project and single-cell RNA-sequencing data from the adult mouse central and peripheral nervous systems^30^. We found enrichment of OCD GWAS signals in six of 13 human brain tissue types in GTEx but no enrichment in human peripheral tissues (Fig. 2b and Supplementary Table 15). In the adult mouse central and peripheral nervous systems, we found enrichment of OCD GWAS signals in 41 of 166 tested specific single cell types using the MAGMA gene set enrichment test (Supplementary Table 16). When summarizing results of individual single cell types into groups of cell types defined by the same region or tissue and cell type, nine of 35 were enriched for OCD GWAS signals (top 20 shown in Fig. 2c). Strong enrichment of OCD GWAS signal was especially observed in excitatory neurons of the hippocampus and the cerebral cortex as well as in D_1_ and D_2_ medium spiny neurons (MSNs).

### Genetic relationship of OCD with other phenotypes

Using phenome-wide association analysis, we examined whether the 30 independent OCD-associated loci identified by our GWAS meta-analysis have previously been associated with other phenotypes (see Supplementary Tables 17a–d for lookups in four, partially overlapping GWAS databanks and Table 1 for highlighted associations). We found that 22 of the 30 loci were associated with other phenotypes, including schizophrenia (seven loci), depression and major depressive disorder (two loci), bipolar disorder (one locus), neuroticism (seven loci), educational attainment (seven loci) and body fat mass or body mass index (eight loci).

**Fig. 3:**
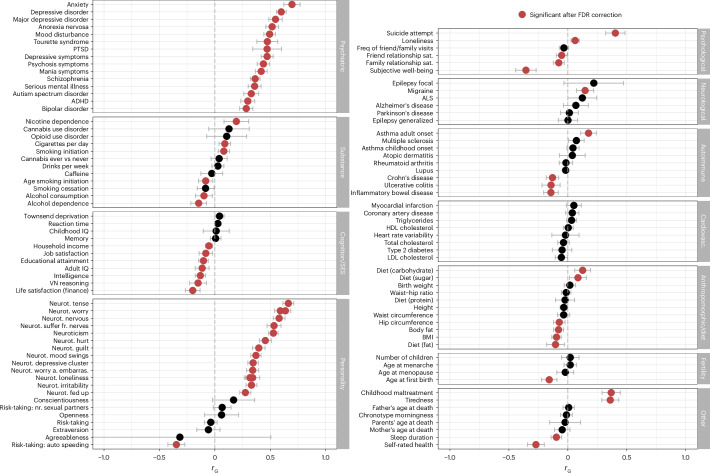
Genetic correlations (*r*_G_) between OCD and 112 phenotypes. This includes psychiatric, substance use, cognition–socioeconomic status (SES), personality, psychological, neurological, autoimmune, cardiovascular (cardiovasc.), anthropomorphic–diet, fertility and other phenotypes. References and sample sizes of the corresponding summary statistics of the GWAS studies can be found in Supplementary Table 18. The OCD summary statistics are of the main meta-analysis (*n*_cases_ = 53,660 and *n*_controls_ = 2,044,417). Error bars represent the 95% confidence intervals for the genetic correlation estimates (*r*_G_). Red circles indicate significant associations with *a P* value adjusted for multiple testing with the Benjamini–Hochberg procedure to control the FDR (<0.05). Black circles indicate associations that are not significant. a., after; ADHD, attention-deficit hyperactivity disorder; ALS, amyotrophic lateral sclerosis; BMI, body mass index; embarras., embarrassment; freq, frequency; fr., from; HDL, high-density lipoprotein; IQ, intelligence quotient; LDL, low-density lipoprotein; neurot., neuroticism; nr., number; PTSD, post-traumatic stress disorder; sat., satisfaction; VN, verbal-numerical.

We further used bivariate linkage disequilibrium score regression (LDSC)^31^ to investigate the extent of genetic correlations between OCD and 112 previously published GWASs encompassing psychiatric, substance use and neurological phenotypes, among others (Fig. 3). We found that 65 phenotypes were significantly correlated with OCD after correcting for multiple testing using the Benjamini–Hochberg^32^ procedure to control the false discovery rate (FDR) at a threshold of 0.05. OCD was significantly positively correlated with all tested psychiatric phenotypes; the highest correlations were with anxiety (${r}_{\rm{G}}=0.70$), depression (${r}_{\rm{G}}=0.60$), anorexia nervosa (${r}_{\rm{G}}=0.52$), Tourette syndrome (${r}_{\rm{G}}=0.47$) and post-traumatic stress disorder (PTSD; ${r}_{\rm{G}}=0.48$). Significant positive genetic correlations were also obtained for neuroticism (${r}_{\rm{G}}=0.53$), in particular for the worry subcluster (${r}_{\rm{G}}=0.64$), and all individual items in the worry subcluster, with slightly lower estimates for the depressive subcluster (${r}_{\rm{G}}=0.35$). Suicide attempt (${r}_{\rm{G}}=0.40$), history of childhood maltreatment (${r}_{\rm{G}}=0.37$) and tiredness (${r}_{\rm{G}}=0.36$) were also notable for strong positive associations with OCD. Of the assessed neurological disorders, OCD was only significantly correlated with migraine (${r}_{\rm{G}}=0.15$). Some autoimmune disorders, such as Crohn’s disease (${r}_{\rm{G}}=-0.13$), ulcerative colitis (${r}_{\rm{G}}=-0.14$) and inflammatory bowel disease (${r}_{\rm{G}}=-0.14$), showed negative correlations with OCD (see Fig. 3 and Supplementary Table 18 for all genetic correlation estimates, 95% confidence intervals and *P* values, Supplementary Note 6 for a more in-depth discussion of all significant genetic correlations and Supplementary Table 19 and Supplementary Figs. 41 and 42 for subgroup-specific genetic correlation estimates).

## Discussion

The OCD GWAS reported here, comprising over 53,000 cases, identified 30 independent genome-wide significant loci. Common SNPs explained 6.7% of the variation in OCD risk in our meta-analysis (LDSC with an assumed population prevalence of 1%), a significant reduction from the 28% reported previously^10^. However, differences in the assumed population prevalence (where a lower assumed prevalence for LDSC heritability calculation results in a lower heritability estimate) and an increase in sample heterogeneity likely contributed to this discrepancy. The reduction in SNP heritability is in line with previous observations for closely related psychiatric disorders such as attention deficit hyperactivity disorder (ADHD)^33,34^ or depression^17,35–37^, where expanding the phenotype definition increased genetic heterogeneity, potentially accounting for the observed decrease in SNP heritability. This aligns with the fact that heritability estimates for more homogeneous OCD subgroups were higher: 16.4% for the clinically ascertained subgroup and 13.3% for the comorbid subgroup (Supplementary Note 10). The current estimates are comparable to those of other psychiatric and substance use disorders, with SNP heritability estimates ranging between 9% and 28%^38^.

The most significant SNP (rs78587207 ($P=5.28\times 1{0}^{-12}$)) identified in the GWAS is located on chr11q12.1 and has been previously associated with several traits, including neuropsychiatric phenotypes^39^ such as depressive symptoms^40^ and neuroticism^40^. Gene-based analyses identified four putative causal genes within this locus. The closest gene to rs78587207 is *CTNND1*, which encodes the cell adhesion molecule p120 catenin. This gene was associated with OCD using three gene-based tests (mBAT-combo, TWAS and PWAS), and we found strong evidence for colocalization of the TWAS signal for *CTNND1* in the dlPFC. The dlPFC has been consistently implicated in the neural circuitry of OCD as well as in compulsivity more broadly as part of the cortico–striatal–thalamo–cortical circuitry^41,42^. The protein product of *CTNND1* is a regulator of cell–cell adhesion^43^ and has a crucial role in gene transcription, Rho GTPase activity and cytoskeletal organization^44–46^. Other credible causal genes in the locus include *CLP1* (cleavage factor polyribonucleotide kinase subunit 1), *TMX2* (thioredoxin-related transmembrane protein 2) and *ZDHHC5* (zinc finger DHHC type palmitoyltransferase 5). Rare genetic mutations in *CLP1* are associated with pontocerebellar hypoplasia type 10, a very rare autosomal recessive neurodegenerative disease characterized by brain atrophy and delayed myelination resulting in intellectual disability^47^. *TMX2* is associated with increased risk of neurodevelopmental disorders with microcephaly, cortical malformations, spasticity and congenital nervous system abnormalities^48^. *ZDHHC5* is broadly expressed in the brain, including the frontal cortex. *ZDHHC5* has not been implicated in brain development but has been linked to lung acinar adenocarcinoma and lung papillary adenocarcinoma in prior studies^49^.

Our finding that approximately 11,500 (s.e. = 607) causal variants account for 90% of the SNP-based heritability of OCD suggests that OCD is more polygenic than other complex traits such as height (*n*_causal_ = 4,000), schizophrenia (*n*_causal_ = 9,600) and ADHD (*n*_causal_ = 5,600) but less polygenic than major depression (*n*_causal_ = 14,500) and educational attainment (*n*_causal_ = 13,200)^50^.

We identified a total of 25 credible causal genes based on robust evidence using multiple positional and functionally informed gene-based approaches. Notably, *DLGAP1*, which has been previously implicated in OCD pathogenesis^10,51^, was not identified in either the GWAS or in the gene-based analyses. Of the 25 genes that were implicated, 15 were within 6.5 kb of a SNP that surpassed genome-wide significance in the meta-analysis. In addition to the four genes discussed above, several others are of particular interest, including *WDR6* and *DALRD3*, which had the strongest evidence from the gene-based analyses. These genes lie in a gene-rich region on chr3p21.31, which, in addition to harboring multiple genome-wide significant SNPs, has been previously associated with a broad range of psychiatric disorders and related traits, including schizophrenia^39^, well-being^52^ and the worry subcluster of neuroticism^53^.

*WDR6* is broadly expressed in the brain, particularly the hypothalamus. Its protein product is involved in cell growth arrest^54^, and recent studies have implicated it in anorexia nervosa^55^ and Parkinson’s disease^56^. *DALRD3* is located on chromosome 3 in the same region as *WDR6. DALRD3*, when fully disrupted, is implicated in a form of epileptic encephalopathy with associated developmental delay^57^. Finally, a third gene in the 3p21 locus, *CELSR3* (cadherin EGF LAG seven-pass G type receptor 3), encodes a protocadherin that is highly expressed in the developing basal ganglia^58^. Multiple loss-of-function mutations in *CELSR3* have been associated with Tourette syndrome^59,60^, which co-occurs with OCD in 10–20% of patients.

Four other genes identified through these analyses are located in the MHC locus, a region on chromosome 6 that has a major role in the adaptive immune system and has been repeatedly linked to major psychiatric disorders^61^. The newly identified MHC association for OCD is noteworthy given evidence linking OCD with autoimmune disorders^62–64^. Genetic pleiotropy may underlie this connection, with variants predisposing individuals to both autoimmune conditions and OCD^65^. Furthermore, some OCD subtypes, such as pediatric acute-onset neuropsychiatric disorders associated with *Streptococcus* and pediatric acute-onset neuropsychiatric syndrome, may have autoimmune origins^66,67^. Nevertheless, we were surprised to discover several negative genetic correlations between OCD and autoimmune disorders such as Crohn’s disease, ulcerative colitis and inflammatory bowel disease in our analyses, suggesting that there is heterogeneity (and perhaps pleiotropy) in the genetic relationships between autoimmune disorders and OCD.

Tissue and cell type enrichment analysis revealed significant enrichment of OCD SNP heritability in several tissues and cell types, with the strongest enrichment in excitatory neurons of the hippocampus and the cerebral cortex and in dopamine D_1_ receptor (D_1_R)-positive and dopamine D_2_ receptor (D_2_R)-positive MSNs in the striatum. These findings are in line with traditional neural circuitry models of OCD, which focus on frontal cortical–striatal pathways^68,69^. These findings are consistent with and build on previous work linking various neuronal cell types to psychiatric and cognitive phenotypes^70^.

Interestingly, the frontal and anterior cingulate cortices, which were enriched in our tissue-based analyses, as well as the hippocampus and the striatum, which were implicated in our cell type-based analyses, are among the regions that are consistently implicated in neuroimaging studies of OCD^41,71–73^. Enrichment in MSNs in the striatum is consistent with their role in the observed aberrant circuitry in OCD, where the D_1_ MSNs project to the globus pallidus interna and the substantia nigra in the direct pathway and the D_2_ type MSNs project to the globus pallidus externa in the indirect pathway^74^. However, MSNs are also enriched in major depressive disorder^75^, schizophrenia^76^ and intelligence^77^, suggesting that the observed enrichment is not specific for OCD.

Our analyses of the shared genetic risk between OCD and other psychiatric disorders provides further insights into the etiology of OCD. In line with previous observations^38,78^, OCD was significantly genetically correlated with multiple psychiatric disorders and traits. The strongest genetic correlations were observed for anxiety disorders, depression and anorexia nervosa, all of which are highly comorbid with OCD^79^. This aligns with previous findings from cross-disorder analyses suggesting a shared genetic susceptibility among most psychiatric disorders^38,80,81^. A notable exception is our finding that risk variants for OCD are protective for alcohol dependence^82^, which is at odds with epidemiological evidence strongly linking OCD and alcohol-related disorders^83^ but in line with a recent paper^79^ reporting a lower-than-expected lifetime comorbidity of substance use disorders in OCD. The observed pattern of correlations with other phenotypes can be thought of as falling into two categories: compulsivity–impulsivity and rumination–worry–neuroticism. In both categories, the patterns of genetic correlations appear to follow a gradient across disorders and traits. For example, in the compulsivity–impulsivity category, strong positive correlations are seen with anorexia nervosa and Tourette syndrome, which are disorders with strong compulsive features, with less positive associations seen with ADHD and negative correlations with alcohol dependence and risk-taking behaviors, which are all phenotypes characterized by impulsivity. A similar gradient is observed for the rumination–worry–neuroticism-related phenotypes, with strong positive correlations with anxiety and other ruminative phenotypes such as worry, transitioning to less strong correlations with individual depression-related items.

This study marks the transition from the flat (sample-building) phase of SNP discovery described for GWAS^84^ (Supplementary Fig. 20), where few to no genome-wide significant loci are identified^10,12,51,85^, to the linear phase of SNP discovery, where even relatively small increases in sample size identify additional genome-wide significant loci^18^. The strengths of the current study therefore include the marked increase in the number of OCD cases and the rigorous analytic methods, including two multivariate approaches (multi-trait analysis of GWAS (MTAG) and GenomicSEM) to control for potential overlapping study participants and to examine potential heterogeneity between the multiple ascertainment approaches. Potential weaknesses include the inability to document comorbid psychiatric disorders in the majority of cases that were not ascertained from clinical collections or electronic registries, the lack of inclusion of non-European ancestries and the limited availability of sex chromosome data. Owing to the nature of our study, imputation references used in the different cohorts were heterogeneous and did not allow for confident analysis of rare variant associations. Future larger-scale sequencing studies that are currently underway will be needed to identify associations in this allele frequency spectrum. We also note that the genetic correlation analyses are impacted by residual heterogeneity in genetic signals owing to the employment of heterogeneous ascertainment strategies.

In summary, this work substantially advances the field of OCD genetics by identifying new OCD genetic risk loci and multiple credible candidate causal genes, including those expressed in brain regions and cell types previously implicated in OCD^86^. We have also shown that OCD is highly polygenic in nature, with many variants implicated not only in OCD but also in commonly comorbid disorders or traits, in particular, anxiety, neuroticism, anorexia nervosa and depression. The observation that common variants explain only a modest amount of the phenotypic variation in OCD suggests that other types of genetic variation may also contribute to the etiology of OCD. Notably, whole-exome-sequencing studies have suggested that a substantial proportion of OCD cases (22%) may be influenced by rare de novo coding variants^87^, especially in genes that are intolerant to loss of function^88^. Similarly, rare potentially damaging copy number variations represent part of the risk architecture for OCD^9^. These findings emphasize the need for a comprehensive exploration of the contribution of both common and rare genetic factors as well as their interplay to OCD risk. Finally, with the implication of the MHC complex, we provide additional evidence for potential shared genetic influences underlying both OCD and increased liability to autoimmune processes, although the directionality of those relationships remains to be definitively elucidated. In addition to continuing to increase sample sizes, future studies will require ancestrally diverse samples to further facilitate the discovery of additional OCD risk variants. Similarly, sex-specific analyses and additional clinical phenotyping will allow for the further elucidation of genetic and clinical relationships between OCD and co-occurring disorders. Finally, with the emergence of drug databases describing the relations between drugs and molecular phenotypes^89^, our results may be useful for drug repurposing (that is, identifying existing drugs targeting OCD risk genes), leading to new opportunities to find more effective treatments.

## Methods

### Ethics

All relevant ethics approvals have been obtained by the respective cohort’s institutions, and a list of all respective approvals can be found in Supplementary Note 2.

### Study participants

We analyzed genomic data from 28 OCD case–control cohorts including 53,660 OCD cases and 2,044,417 controls of European ancestry. Supplementary Table 1 provides an overview of the individual cohorts. A subset of the cases and controls have been included in previous studies^10,51,85^ and preprints^12,13^, as described in Supplementary Note 2. Among all included individuals, 323 cases were part of a parent–proband trio; in these cases, parents were used as pseudocontrols. A total of 20,427 cases met DSM-5 (ref. ^93^) or ICD-10 (https://icd.who.int/) criteria for OCD as assessed by a healthcare professional or derived from (electronic) health records, while the remaining 32,233 cases were based on self-reported OCD diagnosis (23andMe, AGDS and parts of UKBB). Cohort-specific sample and analytic details can be found in Supplementary Note 2. Data collections were approved by the relevant institutional review boards at all participating sites, and all participants provided written informed consent.

### Individual GWAS analyses and harmonizing of results

First, the data of each participating cohort were analyzed individually (see Supplementary Note 2 for details). Genetic data were imputed using either the Haplotype Reference Consortium (HRC)^94^ or 1000 Genomes Project Phase 3 reference panels^95^. The resulting GWAS summary statistics were then harmonized before a conjoint meta-analysis of all autosomes was conducted. Each summary statistic dataset was transformed to the ‘daner’ file format following RICOPILI^96^ specifications. All variants had to meet the following criteria for inclusion: minor allele frequency (MAF) > 1% in cases and controls, INFO score > 0.8 and <1.2. If the effect measure, *P* value or s.e. was missing or was out of bounds (infinite), the SNP was removed. Once cleaned summary statistics were produced, all datasets were aligned to the HRC reference panel. If variants were reported on different strands, they were flipped to the orientation in the HRC reference. Furthermore, strand-ambiguous A/T and C/G SNPs were removed if their MAF was >0.4. In the case that A/T and C/G SNPs showed a MAF < 0.4, allele frequencies were compared to frequencies in the HRC reference. If an allele frequency match was found, that is, minor alleles were the same in the summary statistics and the HRC reference, the same strand orientation was assumed. If an allele mismatch was found, that is, the allele had a frequency > 0.5 in the HRC reference, it was assumed that alleles were reported on different strands, and alleles were flipped subsequently. Marker names were uniformly switched to those present in the HRC reference. If a variant did not overlap with the variants in the HRC reference, it was removed.

### GWAS meta-analysis

Inverse-variance-weighted meta-analysis was conducted on 28 European cohorts using METAL^97^. Weighting was based on standard error primarily to account for the large case–control imbalances in cohorts that used linear mixed model approaches in their primary GWAS. Heterogeneity was assessed with Cochran’s *Q* statistic and the *I*^2^ statistic^98,99^ (see Supplementary Note 5 for details). The genomic control factor lambda (*λ*) was calculated for each individual GWAS and for the overall meta-analysis to identify residual population stratification or systematic technical artifacts. GWAS summary statistics were subjected to LDSC analyses on high-quality common SNPs (INFO score > 0.9) to examine the LDSC intercept to distinguish polygenicity from other types of inflation and to estimate the genetic heritability from the meta-analysis and genetic correlations between cohorts. The genomic inflation factor *λ* was estimated at 1.330 with a *λ*_1000_ of 1.033, while the LDSC intercept was 1.0155 (s.e. = 0.0085), indicating that the inflation was mostly due to polygenic signal and unlikely to be substantially confounded by population structure. The genome-wide significance threshold for the GWAS was set at *a P* value of $5.0\times 1{0}^{-8}$. The 23andMe data included information on the X chromosome; as this information was not present for all other cohorts, analysis of the X chromosome was only conducted in this subcohort (see Supplementary Note 4 for details).

We further conducted GWAS meta-analyses on the following four subgroups, defined by differences in their sample ascertainment: (1) clinical OCD cases diagnosed by a healthcare professional in a clinical setting (*n*_cases_ = 9,089, *n*_controls_ = 21,077; including IOCDF, IOCDF_trio, EPOC, NORDiC-nor, NORDiC-swe, EGOS, OCGAS, OCGAS-ab, OCGAS-gh, OCGAS-nes, Psych_Broad, WWF, MVP, Michigan/Toronto IGS, YalePenn, Chop, CoGa), (2) comorbid individuals who were primarily ascertained for another comorbid psychiatric disorder (*n*_cases_ = 5,266, *n*_controls_ = 43,760; AGDS, iPSYCH), (3) biobank data from large-scale biobanks or registries with ICD or DSM codes (*n*_cases_ = 9,138, *n*_controls_ = 1,049,776; BioVU, EstBB, FinnGen, HUNT, MoBa, UKBB) or (4) 23andMe data (*n*_cases_ = 30,167, *n*_controls_ = 929,804). While these groups are not exclusive (for example, diagnoses in health records were originally given in a clinical setting or comorbid cases were also assessed in a clinical setting or derived from health records), we defined these groups by the cohort’s primary characteristic. We also conducted one meta-analysis including all clinical, comorbid and biobank subgroups, while excluding the 23andMe data, resulting in 23,493 cases and 1,114,613 controls. As 23andMe is the only consumer-based dataset, we intended to compare this dataset to all others.

### Number of trait-specific causal variants (MiXeR analysis)

We applied MiXeR version 1.3 (ref. ^15^) to quantify the polygenicity of OCD (that is, estimate the total number of trait-influencing genetic variants). MiXeR fits a Gaussian mixture model assuming that common genetic effects on a trait are a mixture of causal variants and noncausal variants. Polygenicity is reported as the number of causal variants that explain 90% of SNP heritability of OCD (to avoid extrapolating model parameters into the area of infinitesimally small effects).

### SNP-based fine-mapping (GCTA-COJO)

We performed a conditional and joint analysis (GCTA-COJO)^14^ to identify independent signals within significant OCD loci. This approach performs a conditional and joint analysis on the basis of conditional *P* values before calculating the joint effects of all selected SNPs. We used the stepwise model selection procedure to select independently associated SNPs. The linkage disequilibrium reference sample was created from 73,005 individuals from the QIMR Berghofer Medical Research Institute genetic epidemiology cohort. The distance assumed for complete linkage disequilibrium was 10 Mb, and we used the default *P*-value threshold of $5\times {10}^{-8}$ to define a genome-wide significant hit.

### Multi-trait analysis of ascertainment subgroups

We used MTAG^100^ to conduct multivariable GWAS analyses, reporting GWAS results for each of the ascertainment-specific subgroups. Through this approach, we aimed to address potential concerns about heterogeneity in genetic liability for individual subgroups following different ascertainment strategies. MTAG is a multi-trait analysis that is usually used to combine different but related traits into one meta-analysis by leveraging the shared heritability among the different traits and thereby gaining power. In this case, our aim was to generate ascertainment-specific estimates, while boosting power by leveraging the high shared heritability between the subgroups. The MTAG analysis resulted in four different GWAS summary statistics, one for each subgroup (clinical, comorbid, biobanks, 23andMe). We performed maxFDR analyses to approximate the upper bound on the FDR of MTAG results.

### GenomicSEM

Similarly, we used GenomicSEM^16^ to model the joint genetic architecture of the four subgroups. First, we ran a common factor model without individual SNP effects, following the tutorial ‘Models without individual SNP effects’ on the GenomicSEM GitHub website (Code availability). Second, we ran a multivariate GWAS of the common factor (see Supplementary Note 5 for details). We specified the model using unit variance identification, for which the latent factor variance is fixed to 1 and the loadings of the traits are estimated freely. This ensures that we capture how much of each subgroup contributes to the latent factor. GenomicSEM also generates ${Q}_{{\rm{SNP}}}$ values, which indicate possible heterogeneous effects across the subgroups. The ${Q}_{{\rm{SNP}}}$ statistic is mathematically similar to the *Q* statistic from standard meta-analysis and is a ${ X}^{2}$-distributed test statistic, with larger values indexing a violation of the null hypothesis that the SNP acts entirely through the common factor.

### SNP heritability estimation

The proportion of the phenotypic variance that could be explained by the aggregated effect of all included SNPs (SNP-based heritability, ${h}_{{\rm{SNP}}}^{2}$) was estimated using LDSC^31^. The analysis was performed using precomputed linkage disequilibrium scores from samples restricted to European ancestry in the 1000 Genomes Project^95^, filtered for SNPs included in the HapMap 3 reference panel^101^. SNP heritability was estimated based on the slope of the LDSC, with heritability on the liability scale calculated assuming a 1% population prevalence of OCD^1^. To omit a downward bias in our estimates of liability-scale heritability, following Grotzinger et al.^102^, we accounted for varying levels of ascertainment across cohorts in our meta-analysis by summing the effective sample sizes across the contributing cohorts and using that as the input sample size for LDSC. For conversion to the liability scale (1%), the sample prevalence was then specified as 0.5. The SNP heritability was calculated for the whole OCD sample as well as for ascertainment-specific subgroups.

### Genetic correlations

We used cross-trait LDSC^31^, a method that computes genetic correlations between GWASs without bias from ancestry differences or sample overlap to calculate genetic correlations between the primary OCD meta-analysis and other phenotypes of interest. The selection of traits was based on phenotypic relevance and/or prior report of a genetic relationship with OCD. The genetic correlation between traits was based on the estimated slope from the regression of the product of *Z* scores from two GWASs on the linkage disequilibrium score and represents the genetic covariation between two traits based on all polygenic effects captured by the included SNPs. The genome-wide linkage disequilibrium information used by these methods was based on European populations from the HapMap 3 reference panel^101^, and GWAS summary statistics were filtered to only include SNPs that were part of the 1,290,028 HapMap 3 SNPs.

To ensure the internal consistency of the datasets included in our meta-analysis, we calculated genetic correlations between all cohorts we considered to have a sample size large enough for LDSC (effective sample size of ≥1,000) and between the four ascertainment-specific subgroups.

We further calculated genetic correlations between OCD and 112 other disorders and traits. The source studies of the GWAS summary statistics can be found in Supplementary Table 18. As a follow-up, we also calculated genetic correlations between the 112 phenotypes and each ascertainment-specific subcohort and compared the genetic correlation patterns between the four groups. For all cross-phenotype genetic correlation analyses, we adjusted *P* values for multiple testing using the Benjamini–Hochberg procedure to control for the FDR (<0.05).

### Gene-based analyses

To match the significant SNPs to the genes for which they likely influence function, we conducted a series of positional and functional gene-mapping analyses. The positional mapping employed MBAT-combo^19^, while the functional mapping tested whether genetic variants associated with OCD were also associated with differential expression of nearby genes (within a 1-Mb window) using (1) TWAS^20^ using PsychENCODE data and included colocalization with COLOC^27,28^, and (2) SMR^22^ using whole-blood eQTL information and brain tissues from MetaBrain, alongside the HEIDI test, which tests for heterogeneity in GWAS signal and eQTL association. Furthermore, a PWAS was conducted. As a final step, genes within each locus were prioritized using PsyOPS^26^, which integrates both positional and functional information. The details of each method are described below.

### Positional gene mapping (MBAT-combo)

A gene-based analysis was conducted using mBAT-combo^19^ within GCTA version 1.94.1 (ref. ^14^). The European subsample (*n* = 503 individuals) from phase 3 of the 1000 Genomes Project^95^ was used as the linkage disequilibrium reference panel with the fastBAT default linkage disequilibrium cutoff of 0.9 applied. After filtering SNPs with MAF > 0.01, there were 6,629,124 SNPs for analysis in our sample. A gene list consisting of 19,899 protein-coding genes was used to map the base pair position of genes using genome build hg19 (see Supplementary Note 7 for details).

### Functional gene mapping

#### Transcriptome-wide association study

We used TWAS FUSION^20^ to perform a TWAS of OCD. We used brain gene expression weights from the PsychENCODE^103^ and linkage disequilibrium information from the 1000 Genomes Project Phase 3 (ref. ^95^). TWAS FUSION uses reference linkage disequilibrium and reference gene expression panels with GWAS summary statistics to estimate the association between gene expression and OCD risk. These data were processed with the test statistics from the OCD GWAS to estimate the expression–GWAS association statistic. We corrected for multiple testing using Bonferroni correction.

We performed colocalization analyses using the COLOC R function^27,28^ implemented in TWAS FUSION. Colocalization is a Bayesian method used to calculate the posterior probabilities (PP) that individual lead SNPs within a significant TWAS locus are (1) independent (for example, two causal SNPs in linkage disequilibrium, one affecting transcription and one affecting OCD; PP_3_) or (2) share the same associated variant (for example, a single causal SNP affects both transcription and OCD (PP_4_)). We also performed a conditional analysis to determine whether identified associations represented independent associations. This was performed using the FUSION software, which jointly estimates the effect of all significant features within each locus by using residual SNP associations with OCD after accounting for the predicted expression of other features.

#### Summary-based Mendelian randomization

SMR^22^ was performed using default settings and eQTL meta-analysis summary statistics from European populations for whole blood from eQTLGen^23^ and all five nervous system tissues from MetaBrain (basal ganglia, cerebellum, cortex, hippocampus and spinal cord)^104^. The HEIDI test was performed alongside SMR to test for effect size heterogeneity between the GWAS and eQTL summary statistics. Both SMR and TWAS have a number of important assumptions and limitations, which we discuss in Supplementary Note 9.

### Psychiatric omnilocus prioritization score

We used the gene prioritization method PsyOPS^26^ to rank genes within genome-wide significant loci. This supervised approach integrates biological annotations on mutational intolerance, brain-specific expression and involvement in neurodevelopmental disorder for genes within significant loci. Genes with the top PsyOPS score within each locus were used for further gene prioritization (Gene prioritization). In the instance where two genes in the same locus had the same PsyOPS score, the gene nearest the index SNP was prioritized.

### Protein-wide association study

We performed a PWAS using protein expression data from human brain samples. Human brain proteome reference weight data were obtained using the Religious Orders Study and Rush Memory and Aging Project (ROS/MAP) and the Banner Sun Health Research Institute (Banner) study. The ROS/MAP proteomes were generated from the dlPFC of 376 participants of European ancestry and included 1,476 proteins with significant SNP-based heritability (*P* < 0.01). The Banner PWAS weights were generated from 152 individuals of European ancestry and included 1,147 proteins with significant SNP-based heritability. The PWAS was performed using the TWAS FUSION software^20^ with linkage disequilibrium reference information from the 1000 Genomes Project Phase 3 (ref. ^95^). We corrected for multiple testing using Bonferroni correction.

### Gene prioritization

We created a list of prioritized genes using both gene-based tests and colocalization–HEIDI filters. Results from each gene-based test were first restricted to protein-coding genes with unique gene identifiers based on the release from GENCODE (version 40) for hg19. The following criteria were then used to prioritize genes: (1) a significant (Bonferroni-corrected) association from at least two gene-based tests (mBAT-combo, TWAS FUSION, SMR or PsyOPS) and (2) evidence of colocalization (COLOC PP_4_ > 0.8) and/or significant SMR association with HEIDI *P* > 0.05. Joint–conditional tests of association and significant PWAS associations were used as ancillary approaches to further annotate the prioritized gene list.

### Tissue and cell type enrichment analysis

An analysis of tissue and cell type enrichment of OCD GWAS association signals was conducted using MAGMA (version 1.08)^105^ and partitioned LDSC^106^. We used the previously described approach^29^ to determine gene expression specificity in bulk tissue RNA-seq data from 37 tissues in GTEx (version 8) and single-cell RNA-sequencing data from 19 regions in the mouse central and peripheral nervous systems^30^. The analysis was limited to protein-coding genes with 1:1 orthologs between mice and humans. Gene expression in each tissue or cell type was calculated relative to total expression across all tissues or cell types. Enrichment analysis was performed on genes with the top 10% specificity values in each tissue or cell type, as previously defined^29^.

To evaluate the enrichment of tissue- and cell type-specific genes in OCD genetic association signals, we applied MAGMA and partitioned LDSC. We restricted the analysis to summary statistics for SNPs with a high INFO score (>0.6) and frequency in the entire cohort (MAF > 0.01). Using MAGMA (version 1.08), we tested whether genes with the top 10% specificity in a tissue or cell type showed enrichment in gene-level genetic associations for OCD, with the 1000 Genomes Phase 3 European sample genotypes serving as the linkage disequilibrium reference panel. We used standard gene boundaries (35 kb upstream of the transcription start site to 10 kb downstream of the transcription stop site). Partitioned LDSC was used to examine whether SNPs within 100-kb regions of the top 10% specifically expressed genes were enriched for SNP-based heritability for OCD. All results were corrected for multiple testing with an FDR threshold of 0.05.

### SNP and gene findings in the context of previous analyses

#### Previously reported associations for significant SNPs (PheWAS)

Multiple resources were used to identify previously reported associations of our 30 significant SNPs with other phenotypes. We used the IEU Open GWAS project^92^, PheWAS analysis of GWAS ATLAS^52^ and the NHGRI-EBI GWAS Catalog^91^ and identified credible SNPs through CAUSALdb^90^. CAUSALdb estimates causal probabilities of all genetic variants in GWAS significant loci using three state-of-the-art fine-mapping tools including PAINTOR, CAVIARBF and FINEMAP^107–110^. We used default settings for our CAUSALdb queries.

#### Lookup of previous OCD GWAS findings

We performed a lookup of SNPs identified to be significantly associated with OCD-related phenotypes in previous GWASs. Note that this is not an independent replication, as previous studies partially overlap with the cohorts included in this GWAS.

#### Overlap of previous rare coding variants in OCD and GWAS gene findings

We performed a bidirectional lookup, assessing (1) whether gene findings from our GWAS showed evidence for rare variant involvement and (2) vice versa, whether findings from rare variant testing showed evidence of common variant association in our GWAS.

First, we comprehensively assessed the overlap between 251 genes that we highlighted in our study as carrying common risk variation for OCD (Supplementary Table 14) and current gene-based summary statistics from OCD exome-sequencing data. We used data from Halvorsen et al.^88^ because it is the largest published exome-sequencing study of OCD presently. The supplementary materials from that paper include de novo variant calls from 771 case trios and 1,911 controls (supplementary table 14 in ref. ^88^). We compared the burden of de novo variants, partitioned by variant annotation (synonymous, missense, loss of function) in trio cases versus trio controls within these 251 GWAS-prioritized genes. As described previously^88^, we only included de novo variants that were in loci well covered in both case and control data (In_Jointly_Covered_Loci==TRUE). We also excluded all calls from quartet samples in ref. ^88^ (Cohort!=“OCD_JHU_quartets”). For each of the four variant annotation classes, we compared the proportion of cases that had at least one qualifying de novo variant to the proportion of controls using a two-sided Fisher’s exact test.

Second, as Halvorsen et al.^88^ describe an overall excess of loss-of-function variants in OCD cases relative to controls specifically within loss-of-function intolerant genes (supplementary table 13 in ref. ^88^), we analyzed the overlap between those genes and our GWAS-derived genes. We looked up 200 genes with a probability of loss-of-function intolerance > 0.995 (derived from ref. ^111^) and effect size estimate > 1. We further tested for a difference in the proportion of these pLI > 0.995 genes with effect size estimate > 1 versus ≤1 within the set of genes highlighted in the OCD GWAS (*n* = 251) versus outside this set using a two-sided Fisher’s exact test.

### Reporting summary

Further information on research design is available in the Nature Portfolio Reporting Summary linked to this article.

## Online content

Any methods, additional references, Nature Portfolio reporting summaries, source data, extended data, supplementary information, acknowledgements, peer review information; details of author contributions and competing interests; and statements of data and code availability are available at 10.1038/s41588-025-02189-z.

## Supplementary information

Supplementary Information Supplementary Notes 1–10 and Figs. 1–43

Reporting Summary

Supplementary Tables Supplementary Tables 1–20.

## Data Availability

The meta-analyzed summary statistics (not including 23andMe data) are available from the Psychiatric Genomics Consortium Download page (https://www.med.unc.edu/pgc/download-results/). In line with 23andMe regulations, 10,000 SNPs from the full GWAS including 23andMe are also being made available at https://www.med.unc.edu/pgc/download-results/. The full GWAS summary statistics for the 23andMe discovery dataset will be made available through 23andMe to qualified researchers under an agreement with 23andMe that protects the privacy of the 23andMe participants. Datasets will be made available at no cost for academic use. Please visit https://research.23andme.com/collaborate/#dataset-access/ for more information and to apply to access the data. MVP summary statistics are made available through dbGAP request under accession https://www.ncbi.nlm.nih.gov/projects/gap/cgi-bin/study.cgi?study_id=phs001672.v12.p1 phs001672.v12.p1.

## Code availability

Core analysis code for RICOPILI can be found at https://sites.google.com/a/broadinstitute.org/ricopili/. This includes PLINK (https://www.cog-genomics.org/plink2/), EIGENSOFT (https://www.hsph.harvard.edu/alkes-price/software/), Eagle2 (https://alkesgroup.broadinstitute.org/Eagle/), Minimac3 (https://genome.sph.umich.edu/wiki/Minimac3), SHAPEIT3 (https://mathgen.stats.ox.ac.uk/genetics_software/shapeit/shapeit.html), METAL (https://genome.sph.umich.edu/wiki/METAL_Documentation) and LDSR (https://github.com/bulik/ldsc). MAGMA can be found at https://ctg.cncr.nl/software/magma. GenomicSEM, specifically the tutorial ‘Models without Individual SNP effects’ can be found here: https://github.com/GenomicSEM/GenomicSEM/wiki/3.-Models-without-Individual-SNP-effects. TWAS FUSION can be found at http://gusevlab.org/projects/fusion/. PWAS: for access to the protein weights, see https://www.synapse.org/#!Synapse:syn24872746. GCTA (mBAT-combo and COJO) can be found at https://yanglab.westlake.edu.cn/software/gcta/#Overview. LDSC and partitioned heritability can be found at https://github.com/bulik/ldsc. Additional code for data processing (for example, harmonization of summary statistics) can be found at 10.6084/m9.figshare.28451894 (ref. ^112^).
