# Supplementary material for: Genome-wide analyses identify 30 loci associated with obsessive–compulsive disorder
Source: Nat Genet. 2025 May 13;57(6):1389–401. doi: 10.1038/s41588-025-02189-z (PMC12165847; doi:10.1038/s41588-025-02189-z)
Supplement: Supplementary file 2 — Reporting Summary [file 41588_2025_2189_MOESM2_ESM.pdf]

nature portfolio

Corresponding author(s): Manuel Mattheisen and Nora Strom

Last updated by author(s): Mar 6, 2025

Reporting Summary

Nature Portfolio wishes to improve the reproducibility of the work that we publish. This form provides structure for consistency and transparency in reporting. For further information on Nature Portfolio policies, see our Editorial Policies and the Editorial Policy Checklist.

Statistics

For all statistical analyses, confirm that the following items are present in the figure legend, table legend, main text, or Methods section.

n/a	Confirmed	
☐	☒	The exact sample size (*n*) for each experimental group/condition, given as a discrete number and unit of measurement
☐	☒	A statement on whether measurements were taken from distinct samples or whether the same sample was measured repeatedly
☐	☒	The statistical test(s) used AND whether they are one- or two-sided<br>*Only common tests should be described solely by name; describe more complex techniques in the Methods section.*
☐	☒	A description of all covariates tested
☐	☒	A description of any assumptions or corrections, such as tests of normality and adjustment for multiple comparisons
☐	☒	A full description of the statistical parameters including central tendency (e.g. means) or other basic estimates (e.g. regression coefficient) AND variation (e.g. standard deviation) or associated estimates of uncertainty (e.g. confidence intervals)
☐	☒	For null hypothesis testing, the test statistic (e.g. *F*, *t*, *r*) with confidence intervals, effect sizes, degrees of freedom and *P* value noted<br>*Give P values as exact values whenever suitable.*
☒	☐	For Bayesian analysis, information on the choice of priors and Markov chain Monte Carlo settings
☒	☐	For hierarchical and complex designs, identification of the appropriate level for tests and full reporting of outcomes
☒	☐	Estimates of effect sizes (e.g. Cohen's *d*, Pearson's *r*), indicating how they were calculated

Our web collection on statistics for biologists contains articles on many of the points above.

Software and code

Policy information about availability of computer code

Data collection	No software was used for data collection. For individual studies please refer to their respective publications.
Data analysis	This is fully described in the Online Methods and associated Supplementary Information. In brief: Additional quality control,a ssociation analyses,a nd polygenic risk scoring was done either using the Ricopili (v 1118b) pipeline: https://github.com/Nealelab/ricopili,w hich relies on the following software: Eigensoft 6.0.1 (incl. smartPCA),P link 1.9,M ETAL 2011-03-25 or in other samples with comparable software tools and approaches (more details can be found in the Supplemental Table 51 and the Supplemental Text.<br>SNP-based finemapping was performed using GCTA-COJO (as part of the GCTA vl.94.1 release),M TAG vl.08 was used for multivariable GWAS or the respective subgroups. Genomic SEM v 0.04 was used to run structural equation analsyes,L DSC (v 1.0.1; https://github.com/bulik/ldsc) was used to calculate all heritability estimates and genetic correlations.Gene-based analyses were performed using MBAT-COMBO (within the GCTA package vl.94.1),TWAS Fusion (http://gusevlab.org/projects/fusion/; accessed Feb 2023),SMR v 1.3.1 (https://yanglab.westlake.edu.cn/software/smr/#Overview).<br>Tissue and cell-type enrichment of OCD GWAS association signals was conducted using MAGMA (vl.08).<br>R v3.4 was used in general for statistical analyzes and plotting (https://www.Rproject.org)<br>MiXER v1.2 was used to calculate the number of causal variants accounting for 90% of the SNP heritability in OCD.<br>Analyses for PGC analyses were conducted on the SurfSara Lisa computing infrastructure and for all other analyses on the Bianca server as part of the Uppsala Multidisciplinary Center for Advanced Computational Science.

For manuscripts utilizing custom algorithms or software that are central to the research but not yet described in published literature, software must be made available to editors and reviewers. We strongly encourage code deposition in a community repository (e.g. GitHub). See the Nature Portfolio guidelines for submitting code & software for further information.

Data

Policy information about availability of data

All manuscripts must include a data availability statement. This statement should provide the following information, where applicable:

- Accession codes, unique identifiers, or web links for publicly available datasets
- A description of any restrictions on data availability
- For clinical datasets or third party data, please ensure that the statement adheres to our policy

The meta-analyzed summary statistics (not including 23andMe data) will be made available via the Psychiatric Genomics Consortium Download page (https://www.med.unc.edu/pgc/download-results/). In line with 23andMe regulations, 10,000 SNPs from the full GWAS incl. 23andMe, will be made available.
The full GWAS summary statistics for the 23andMe discovery data set will be made available through 23andMe to qualified researchers under an agreement with 23andMe that protects the privacy of the 23andMe participants. Datasets will be made available at no cost for academic use. Please visit https://research.23andme.com/collaborate/#dataset-access/ for more information and to apply to access the data.
MVP summary statistics are made available through dbGAP request under accession phs001672.v12.p1.

Research involving human participants, their data, or biological material

Policy information about studies with human participants or human data. See also policy information about sex, gender (identity/presentation), and sexual orientation and race, ethnicity and racism.

Reporting on sex and gender	Sex and Gender based analyses were not performed as a consequence of missing samples from the overall collection. We are currently running such analyses (sex-stratified analyses) in a subset of the overall sample and will report in an upcoming publication on the findings. For most samples the number of female and male cases (and controls) were not available and therefore not reported.
Reporting on race, ethnicity, or other socially relevant groupings	Analysis are limited to European ancestry samples, no race,ethnicity or other socially relevant groupings have been made other than those potentially (hiddenly) associated with our sub-groupings for our main phenotype related ascertainment (i.e., self-report, Biobank, comorbid, clinical groups).
Population characteristics	This is a meta-analysis of existing (in part unpublished) summary statistics. Details for the respective individual studies are provided in the Supplemental material and vary. The most common grouping exists for genotyping batches (especially for the large scale cohort and biobanking studies (including 23andMe).
Recruitment	Details are provided in the Supplemental material and vary for the different studies that are part of the meta-analysis. In brief, samples were either ascertained in clinical setting, as part of larger biobanking efforts, via 23andMe, or as part of studies looking into comorbid phenotypes (such as MDD, ASD, and ADHD).
Ethics oversight	The details of the I RB/oversight body that provided approval or exemption for the research described are given below: were obtained from the Hopkins Medicine Institutional Review Boards. OCGAS-ab: The study was approved by REB at Hospital for Sick Children. Ethics approvals for the OCGAS study were obtained from the Hopkins Medicine Institutional Review Boards. OCGAS-gh: Informed written consent was obtained in all cases by the participants or their parents. The study was approved by the ethical commissions of all involved universities in accordance with the latest version of the Declaration of Helsinki, including an ethical permission granted by the Ethic Committees from Aachen, Wuerzburg, Marburg, Frei burg, and the Cantonal Ethic Commission of Zuerich (Ref. Nr. 39/97, 140/3 and EK: KEK-ZHNr. 2010-0340/3). OCD-WWF: The study was approved by the ethics committee of the University of Wuerzburg, Germany and was conducted according to the ethical principles of the Helsinki Declaration. All patients gave written informed consent prior to participation. Psych_Broad: Both studies have been approved by the Clinical Research Ethics Committee (CREC) of Hospital Universitari Vall d'Hebron. All methods were performed in accordance with the relevant guidelines and regulations and written informed consent was obtained from participant parents before inclusion into the study. UKBB: Research on the UK Biobank is conducted under a generic Research Tissue Bank approval from the UK North West Multi-centre Research Ethics Committee (MREC). This research was approved to be conducted under that approval by the governing Research Ethics Committee of the UK Bio bank. The analyses in this paper were performed under an approved extension to project 16577. Yale-Penn: Participants were recruited from eastern U.S sites and provided written informed consent as approved by the institutional review board at each site.<br>23andMe: Participants provided informed consent and volunteered to participate in the research on line, under a protocol approved by the external AAHRPP-accredited IRB, Ethical & Independent (E&I) Review Services. As of 2022, E&I Review Services is part of Salus IRB (https://www.versiticlinicaltrials.org/salusirb). AGDS: All study protocols were approved by the QIMR Berghofer Medical Research Institute Human Research Ethics Committee. The protocol for approaching participants through the DHS, enrolling them in the study, and consenting for all phases of the study (including invitation to future related studies) and accessing MBS and PBS records was approved by the Ethics Department of the Department of Human Services. BioVU: The Vanderbilt University Medical Center Institutional Review Board oversees BioVU and approved this project (IRB201609). COGA: Institutional review boards at all sites approved the study and all participants provided informed consent. EGOS: Ethical approvals were obtained from the Institutional Review Board (IRB) at the Icahn School of Medicine at Mount Sinai, New York, NY, and the Regional Ethical Review Board in Stockholm. EPOC: The study was in accordance with the revised Declaration of Helsinki and approved by the local ethics committees of the Cha rite University Medicine Berlin and the University Hospital Bonn. Est BB: At recruitment, participants signed a consent allowing follow-up linkage of their electronic health records (EH Rs), thereby providing a longitudinal collection of their phenotypic information. FinnGen: The Ethical Review Board of the Hospital District of Helsinki and Uusimaa approved the FinnGen study protocol Nr. HUS/990/2017. The FinnGen project was approved by Finnish Institute for Health and Welfare (THL), approval numbers THL/2031/6.02.00/2017, amendments THL/341/6.02.00/2018, THL/2222/6.02.00/2018, and THL/283/6.02.00/2019. HUNT:

The HUNT study was approved by the Regional Committee for Medical and Health Research Ethics, Norway (2015/575). IOCDF/OCGAS: This work was approved by the relevant IRBs at all participating sites, and all participants provided written informed consent. iPSYCH: The study was approved by the Regional Scientific Ethics Committee in Denmark and the Danish Data Protection Agency. MVP: The U.S. Department of Veterans Affairs (VA) Million Veteran Program (MVP) is collecting genetic and electronic health record (EHR) data in the U.S with ethical approval given by the Central VA Institutional Review Board (IRB) and site-specific IRBs. All relevant ethical regulations for work with human subjects were followed in the conduct of the study, and informed consent was obtained from all participants. MoBa: The establishment of MoBa and initial data collection was based on a license from the Norwegian Data Protection Agency and approval from The Regional Committees for Medical and Health Research Ethics. The MoBa cohort is now based on regulations related to the Norwegian Health Registry Act. NORDiC-SWE: This study was approved by the Regional Ethics Committee, Stockholm (EPN Stockholm) and the Institutional Review Board (IRB) at the University of North Carolina at Chapel Hill and all subjects provided informed consent. NORDiC-NOR: The NORDiC-NOR study was approved by the Norwegian Regional Committee for Medical and Health Research Ethics
(IRB00001872 REK West) under project number 2018/52 REKVest (Pl: Bjarne Hansen) and project number: 2014/75 REKVest (Pl: Jan Haavik) and all subjects provided informed consent. OCGAS-all: Ethics approvals for the OCGAS study were obtained from the Hopkins Medicine Institutional Review Boards, the Butler Institutional Review Board, the UCLA Institutional Review Boards, the Mass General Brigham Human Research Committee, the Columbia University Institutional Review Boards, and the National Institutes of Health Institutional Review Board (NIH IRB). OCGAS-nestadt: Ethics approvals for the OCGAS study were obtained from the Hopkins Medicine Institutional Review Boards. OCGAS-ab: The study was approved by REB at Hospital for Sick Children. Ethics approvals for the OCGAS study were obtained from the Hopkins Medicine Institutional Review Boards. OCGAS-gh: Informed written consent was obtained in all cases by the participants or their parents. The study was approved by the ethical commissions of all involved universities in accordance with the latest version of the Declaration of Helsinki, including an ethical permission granted by the Ethic Committees from Aachen, Wuerzburg, Marburg, Frei burg, and the Cantonal Ethic Commission of Zuerich (Ref. Nr. 39/97, 140/3 and EK: KEK-ZHNr. 2010-0340/3). OCD-WWF: The study was approved by the ethics committee of the University of Wuerzburg, Germany and was conducted according to the ethical principles of the Helsinki Declaration. All patients gave written informed consent prior to participation. Psych_Broad: Both studies have been approved by the Clinical Research Ethics Committee (CREC) of Hospital Universitari Vall d'Hebron. All methods were performed in accordance with the relevant guidelines and regulations and written informed consent was obtained from participant parents before inclusion into the study. UKBB: Research on the UK Biobank is conducted under a generic Research Tissue Bank approval from the UK North West Multi-centre Research Ethics Committee (MREC). This research was approved to be conducted under that approval by the governing Research Ethics Committee of the UK Bio bank. The analyses in this paper were performed under an approved extension to project 16577. Yale-Penn: Participants were recruited from eastern U.S sites and provided written informed consent as approved by the institutional review board at each site.

Note that full information on the approval of the study protocol must also be provided in the manuscript.

Field-specific reporting

Please select the one below that is the best fit for your research. If you are not sure, read the appropriate sections before making your selection.

☒ Life sciences ☐ Behavioural & social sciences ☐ Ecological, evolutionary & environmental sciences

For a reference copy of the document with all sections, see nature.com/documents/nr-reporting-summary-flat.pdf

Life sciences study design

All studies must disclose on these points even when the disclosure is negative.

Sample size	No sample size calculation was made. Individual studies were selected for inclusion if they had at least an effective samples size of 100. Sample size of the meta-analysis is a result of inclusion of all world-wide available samples of European ancestry at the time of study,
Data exclusions	Within each analyzed cohort we aimed at analyzing genetically homogeneous samples of unrelated individuals. Related individuals were excluded based on Identity by State analyses (pseudo controls were used for trios) and genetic outliers were excluded based on principal component analyses.
Replication	Replication in a narrow sense could not be provided given that the meta-analysis comprised of most if not all samples currently available for research into the genetics of OCD (at the time of analysis). Consistency was checked via different sensitivity analyses using sub-groupings, including genomic SEM analyses, MTAG analyses, internal genetic correlation and other approaches. Our analyses did not reveal a concerning amount of heterogeneity in the samples.
Randomization	This study utilized naturally occurring genetic variation as the experimental condition. Because an individual's genotype is fixed at conception, we could not randomly assign participants to experimental groups. Instead, we examined the association between pre-determined genetic profiles and the observed phenotype of OCD.
Blinding	Blinding is not relevant to the current study. Samples were not allocated to different conditions by the researches, and the phenotype ascertainment process is fully separate from the genotyping process.

Reporting for specific materials, systems and methods

We require information from authors about some types of materials, experimental systems and methods used in many studies. Here, indicate whether each material, system or method listed is relevant to your study. If you are not sure if a list item applies to your research, read the appropriate section before selecting a response.

Materials & experimental systems

n/a	Involved in the study
☒	☐ Antibodies
☒	☐ Eukaryotic cell lines
☒	☐ Palaeontology and archaeology
☒	☐ Animals and other organisms
☒	☐ Clinical data
☒	☐ Dual use research of concern
☒	☐ Plants

Methods

n/a	Involved in the study
☒	☐ ChIP-seq
☒	☐ Flow cytometry
☒	☐ MRI-based neuroimaging

Plants

Seed stocks	*Report on the source of all seed stocks or other plant material used. If applicable, state the seed stock centre and catalogue number. If plant specimens were collected from the field, describe the collection location, date and sampling procedures.*
Novel plant genotypes	*Describe the methods by which all novel plant genotypes were produced. This includes those generated by transgenic approaches, gene editing, chemical/radiation-based mutagenesis and hybridization. For transgenic lines, describe the transformation method, the number of independent lines analyzed and the generation upon which experiments were performed. For gene-edited lines, describe the editor used, the endogenous sequence targeted for editing, the targeting guide RNA sequence (if applicable) and how the editor was applied.*
Authentication	*Describe any authentication procedures for each seed stock used or novel genotype generated. Describe any experiments used to assess the effect of a mutation and, where applicable, how potential secondary effects (e.g. second site T-DNA insertions, mosiacism, off-target gene editing) were examined.*
